# Epidermal Growth Factor Receptor-Dependent Mutual Amplification between Netrin-1 and the Hepatitis C Virus

**DOI:** 10.1371/journal.pbio.1002421

**Published:** 2016-03-31

**Authors:** Marie-Laure Plissonnier, Thomas Lahlali, Maud Michelet, Fanny Lebossé, Jessica Cottarel, Melanie Beer, Grégory Neveu, David Durantel, Birke Bartosch, Rosita Accardi, Sophie Clément, Andrea Paradisi, Mojgan Devouassoux-Shisheboran, Shirit Einav, Patrick Mehlen, Fabien Zoulim, Romain Parent

**Affiliations:** 1 Pathogenesis of Hepatitis B and C - Equipe labellisée LabEx DEVweCAN, INSERM U1052, Centre de Recherche en Cancérologie de Lyon, F-69003 Lyon, France, Université de Lyon, F-69003 Lyon, Université Lyon 1, ISPB, Lyon, F-69622, France, CNRS UMR5286, F-69083 Lyon, France, Centre Léon Bérard, F-69008 Lyon, France; 2 Hospices Civils de Lyon, Service d’Hépatogastroentérologie, F-69001 Lyon, France; 3 Department of Medicine, Stanford University School of Medicine, Stanford, California, United States of America; 4 Department of Microbiology and Immunology, Stanford University School of Medicine, Stanford, California, United States of America; 5 International Agency for Research on Cancer, F-69424 Lyon, France; 6 Division of Clinical Pathology, University Hospital, University of Geneva School of Medicine, Geneva, Switzerland; 7 Apoptosis, Cancer and Development Laboratory - Equipe labellisée ‘La Ligue’, LabEx DEVweCAN, CNRS UMR5286, Centre de Recherche en Cancérologie de Lyon, F-69008 Lyon, France, Université de Lyon F-69003 Lyon, Centre Léon Bérard, F-69008 Lyon, France; 8 Hospices Civils de Lyon, Service d’Anatomie Pathologique, F-69001 Lyon, France; University of Wisconsin-Madison, UNITED STATES

## Abstract

Hepatitis C virus (HCV) is an oncogenic virus associated with the onset of hepatocellular carcinoma (HCC). The present study investigated the possible link between HCV infection and Netrin-1, a ligand for dependence receptors that sustains tumorigenesis, in particular in inflammation-associated tumors. We show that Netrin-1 expression is significantly elevated in HCV+ liver biopsies compared to hepatitis B virus (HBV+) and uninfected samples. Furthermore, Netrin-1 was upregulated in all histological stages of HCV+ hepatic lesions, from minimal liver fibrosis to cirrhosis and HCC, compared to histologically matched HCV- tissues. Both cirrhosis and HCV contributed to the induction of Netrin-1 expression, whereas anti-HCV treatment resulted in a reduction of Netrin-1 expression. In vitro, HCV increased the level and translation of Netrin-1 in a NS5A-La-related protein 1 (LARP1)-dependent fashion. Knockdown and forced expression experiments identified the receptor uncoordinated receptor-5 (UNC5A) as an antagonist of the Netrin-1 signal, though it did not affect the death of HCV-infected cells. Netrin-1 enhanced infectivity of HCV particles and promoted viral entry by increasing the activation and decreasing the recycling of the epidermal growth factor receptor (EGFR), a protein that is dysregulated in HCC. Netrin-1 and HCV are, therefore, reciprocal inducers in vitro and in patients, as seen from the increase in viral morphogenesis and viral entry, both phenomena converging toward an increase in the level of infectivity of HCV virions. This functional association involving a cancer-related virus and Netrin-1 argues for evaluating the implication of UNC5 receptor ligands in other oncogenic microbial species.

## Abstract 2

The dependence receptor ligand Netrin-1 and the hepatitis C virus induce each other's expression, generating a mutual amplification loop with potential implications for the development of hepatocellular carcinoma.

## Author Summary

Viruses and bacteria are implicated in 15%–20% of total cancer occurrences. Hepatitis C virus (HCV) infection is one of the main causative agents of liver cancer. “Dependence receptors” are a class of receptors that auto-activate and trigger apoptosis in the absence of their ligands, and “dependence receptor” ligands such as Netrin-1 are known to be overactivated in cancers, especially in inflammation-driven tumors. In this study, we show that HCV and Netrin-1 are mutual inducers—Netrin-1 expression is increased upon HCV infection and, in turn, the rise in Netrin-1 leads to an increase in the HCV particle infectivity. The effects on HCV infectivity involve the liver cancer-related epidermal growth factor receptor (EGFR), which is known to be a host receptor necessary for HCV entry and which we now show is activated by Netrin-1. Our work, therefore, illustrates a pathogenic positive feedback loop involving HCV, Netrin-1, and EGFR, among other factors, in association with cancer development.

## Introduction

Cancers triggered by microbial oncogenes account for approximately 16% of cancer occurrences [1]. Hepatitis C virus (HCV) is a major etiologic agent of hepatocellular carcinoma (HCC), the fifth most common cancer worldwide [2]. The epidermal growth factor receptor (EGFR) is a host factor for entry of HCV [3, 4] as well as for the influenza virus [5] and adeno-associated virus 6 [6]. EGFR signaling is involved in HCC development [7] and possibly in the resistance to the HCC drug sorafenib [8].

An interesting advance in developmental biology and oncology in the last decade was the discovery of dependence receptors (DRs) [9–13], a class of receptors that auto-activate and trigger apoptosis in the absence of their ligands. One such ligand is Netrin-1. Netrin-1 is a secreted protein that was initially identified as the canonical soluble partner of the uncoordinated receptor-5 (UNC5) DR family in the field of neuroembryogenesis. It inactivates UNC5-mediated intrinsic signals, including cell death, unlike most ligands that exert positive pharmacology on their cognate receptors. Recent data support the implication of Netrin-1 and its main receptors in epithelial tissues and suggested its role in the morphogenesis of “branched” organs [11]. In cancer, the model of dependence receptors predicts that instead of losing Netrin-1 receptors, a second potential selective advantage for tumor cell survival could be an autocrine expression of the ligand that inhibits this receptor. Accordingly, Netrin-1, a reprogramming modulator [14], is upregulated in several cancer types [13,15–18] as well as in cancer-associated inflammatory diseases such as colitis and Crohn’s disease [13,19,20]—for review, see Paradisi and Mehlen [21]. The inflammatory response associated with several epithelial disorders thus appears to play a key role in Netrin-1 induction. As is the case for most viral infections, chronic hepatitis C also bears an important inflammatory component, thought to strongly participate in the worsening of the liver structure and function, which could ultimately result in cancer promotion within hepatocytic compartments.

Taken together, these data argue in favor of the establishment of a more complete landscape regarding the interplay between inflammation and cancer. Long-term infections involving inflammation-inducing oncogenic viruses may represent potential factors for the regulation of Netrin receptors. To our knowledge, data linking such factors and oncogenic viruses are nonexistent. In addition, neither the regulation of the *Netrin-1* transcript nor that of the protein have, so far, been identified in association with HCV via high-throughput studies. We therefore decided to investigate the possible interplay between the DR system and HCV, focusing on Netrin-1.

In this study, we show that Netrin-1 is upregulated by HCV and that it participates in a mutual amplification loop with HCV, in turn leading to an enhancement of viral infectivity. Our results indicate that induction of Netrin-1 by HCV represents an important mechanism by which the virus establishes persistent infection and may contribute to neoplastic transformation.

## Results

### HCV-Dependent Netrin-1 Induction

**Fig 1:**
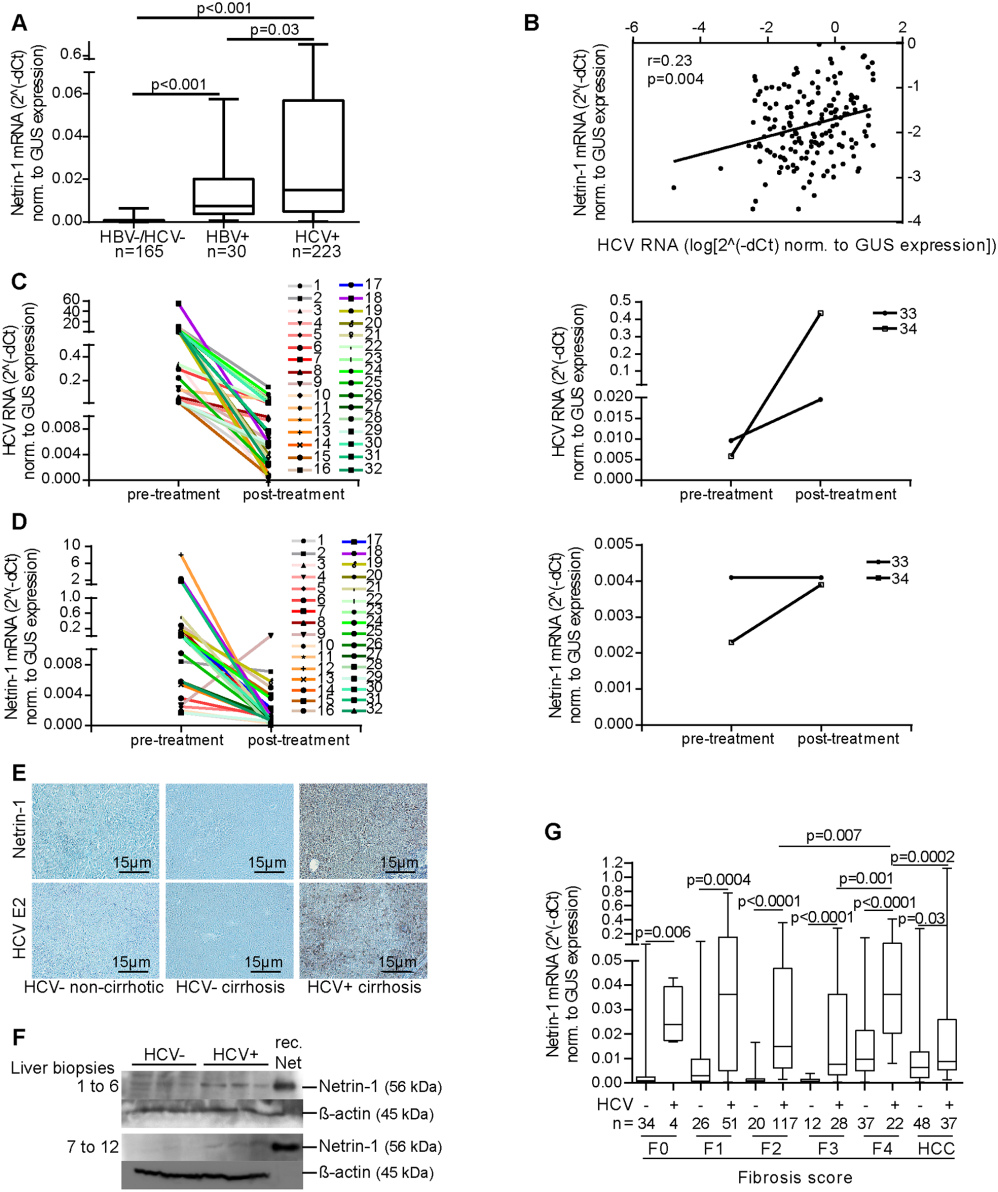
HCV levels correlate with the expression of Netrin-1 in the liver biopsies of HCV-infected patients. **A**. HCV-positive samples exhibit the highest levels of *Netrin-1* mRNA of all the chronic liver disease biopsies. The levels of *Netrin-1* mRNA were quantified by RT-qPCR. Statistical significance was determined using the Mann-Whitney test. **B.** Positive correlation between intrahepatic levels of HCV and *Netrin-1* mRNA. HCV RNA and *Netrin-1* mRNA were quantified by RT-qPCR. Statistical significance was determined using the Spearman test. An outlier test was run to confirm these results. **C and D.** *Netrin-1* mRNA parallels HCV RNA levels upon treatment. HCV RNA and *Netrin-1* mRNA were quantified by RT-qPCR in paired biopsies, before and after treatment, of partially responding patients (**C,D left panels**) and nonresponding patients **(C,D, right panels**). **E.** Parenchymal Netrin-1 staining is associated with HCV infection status in HCV-infected patients. Uninfected, chronic liver disease samples (non-cirrhotic sample, *n* = 1; alcohol-related cirrhosis samples, *n* = 3) and HCV genotype 1-infected cirrhosis samples (*n* = 4) were analyzed. Representative images of Netrin-1 staining (upper panels) and HCV E2 staining (lower panels) are shown. **F.** Netrin-1 protein expression is increased in HCV-positive samples. The level of Netrin-1 was quantified by immunoblotting using recombinant Netrin-1 (rec. Net) as a control. **G.** The levels of *Netrin-1* mRNA are higher in HCV+ *versus* HCV- biopsies, regardless of the histological stage, from normal liver to HCC. Intrahepatic *Netrin-1* mRNA levels were quantified by RT-qPCR. Statistical significance was determined using the Mann-Whitney test, *p* < 0.05. Fibrosis scores were determined by histopathology and using the Fibroscan method [22]. The underlying data for panels in this figure can be found in S1 Data.

HCV, along with several other liver conditions, is known to trigger hepatic inflammation. To establish a connection between the expression of Netrin-1 (Uniprot Acc. # O95631) and viral infection of the liver, we first measured the level of *Netrin-1* mRNA (GenBank Acc. # NM_004822) in 418 liver biopsies, taken either from virus-free patients (165 samples), from HCV-infected patients (223 samples), or from HBV-infected patients (30 samples) (S1 Table). The latter were included as a positive control for chronic viral infection of the liver, and tissue biopsies revealed an 11-fold increase in the level of *Netrin-1* mRNA compared to uninfected controls (Fig 1A). Interestingly, the HCV-infected samples displayed a further 2-fold increase in *Netrin-1* transcripts versus HBV+ samples, totaling a 23-fold increase in *Netrin-1* mRNA levels compared to the uninfected controls. Moreover, a positive correlation was found between the levels of *Netrin-1* mRNA and HCV RNA in those liver biopsies (Fig 1B). Similarly, HCV RNA and *Netrin-1* mRNA levels were measured in patients before and after first-time treatment with interferon and ribavirin, two antiviral compounds, in biopsies obtained from 18 HCV+/HBV- patients. Of these, 16 showed a partial treatment response (i.e., presented a decrease in viral load; Fig 1C, left panel), accompanied in all but one with a clear decrease in *Netrin-1* mRNA levels (Fig 1D, left panel). The two patients who failed to respond to treatment (Fig 1C, right panel) showed stable or increased levels in *Netrin-1* mRNA, which paralleled their stable or increased HCV RNA load (Fig 1D, right panel). These data support the HCV-dependent status of Netrin-1 upregulation in HCV-positive patients.

Chronic HCV infection features gradual worsening of the liver through the replacement of functional hepatocytes by nonfunctional connective tissue, a process called fibrosis. To determine whether an increase in the level of *Netrin-1* mRNA resulted in a concurrent increase at the protein level, immunostaining for Netrin-1 and the HCV E2 envelope glycoprotein antigens was performed on liver samples matched for fibrosis score. Netrin-1 was clearly observed in HCV+ samples compared to their uninfected counterparts, and, furthermore, the use of the well-characterized anti-E2 antibody [23] confirmed that only hepatocytes exhibit positive Netrin-1 staining in infected samples (Fig 1E and S1 Supporting Information). The additional comparison of protein levels in HCV- and HCV+ liver tissues, by immunoblotting, corroborated our findings (Fig 1F). Together, these data indicate that hepatocytes of HCV+ patients express increased levels both of *Netrin-1* mRNA and protein and further strengthen the likelihood that HCV is a hepatocytic Netrin-1 inducer.

It is well known that Netrin-1 induction can result from inflammation, in particular in the gastrointestinal system [21]. To address the specific role of HCV in our model and distinguish fibrosis-associated Netrin-1 induction from HCV-associated Netrin-1 induction, we plotted *Netrin-1* mRNA levels in HCV+ versus HCV- samples against all (F0 to F4/cirrhosis) histological stages. *Netrin-1* mRNA had increased significantly by 25-fold (F0), 15-fold (F1), 17-fold (F2), 12-fold (F3), and 4-fold (F4) in all HCV-infected samples compared to their HCV-uninfected counterparts (HCC: 1.4-fold) (Fig 1G). In addition, levels of *Netrin-1* mRNA were further elevated (>30-fold) in HCV-infected cirrhotic patients compared to control patients (samples F0–F3). Of note, no association could be observed between non-HCV clinical parameters and levels of *Netrin-1* mRNA (S1 Fig; see also S2 Supporting Information for more insight on *Netrin-1* transcript levels in patients). Importantly, a comparison of HCV(-) biopsies revealed that HCV-negative cirrhosis (i.e., F4) samples already displayed the highest 4-fold to 12-fold increase in *Netrin-1* mRNA compared to all other HCV-negative samples (S2C Supporting Information). Taken together, these data suggest that Netrin-1 expression is induced in patients chronically infected with HCV across all stages of the disease, and that HCV and cirrhosis cooperate for higher Netrin-1 induction.

**Fig 2:**
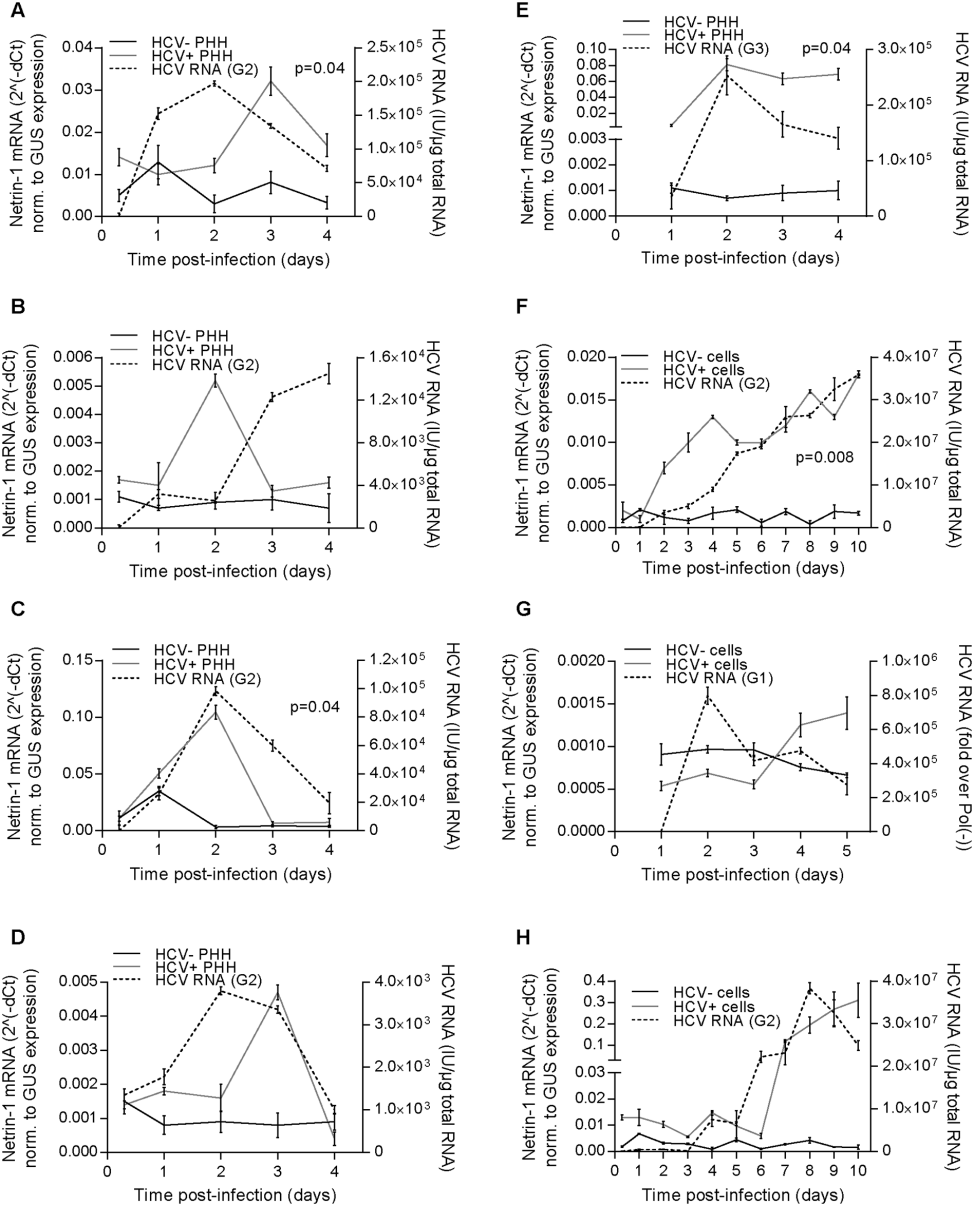
HCV induces the expression of Netrin-1 in vitro. **A, B, C, D.** HCV induces *Netrin-1* mRNA in primary human hepatocytes. Cells were infected at a MOI of 1 with HCV genotype 2 strain 4 d after seeding (*n* = 4 independent preparations from four different patients, Wilcoxon test, *p* < 0.05). **E**. HCV induces *Netrin-1* mRNA in primary human hepatocytes endogenously infected with HCV genotype 3 strain. **F**. HCV induces *Netrin-1* mRNA in Huh7.5 cells. Proliferative Huh7.5 cells were infected at a MOI of 0.1 with HCV genotype 2 strain the day after seeding and trypsinized on day five post-infection. **G**. HCV induces *Netrin-1* mRNA in Huh7.5 cells. Proliferative Huh7.5 cells were electroporated with HCV genotype 1a strain (H77). **H**. Differentiated Huh7.5 cells (right) were incubated with 2% DMSO 4 d after seeding and infected at a MOI of 0.05 3 d after DMSO treatment. The levels of HCV RNA and *Netrin-1* mRNA were quantified by RT-qPCR. Data are represented as mean ± standard deviation (*n* = 3 for each cell phenotype, Wilcoxon test, *p* < 0.05). The underlying data for panels in this figure can be found in S1 Data.

Next, we investigated whether induction of Netrin-1 by HCV could also be seen in vitro, in a tissue inflammation-free environment. Primary human hepatocytes (PHH) were infected with an HCV japanese fulminant hepatitis 1 (JFH1; genotype 2) selected for its higher rate of propagation in cell cultures [24]. Results revealed a peak in *Netrin-1* upregulation (by 5-fold to 20-fold) in the HCV-infected cultures at day two or day three (Fig 2A–2D). This was also confirmed in endogenously infected PHH with wild-type genotype 3 virus (Fig 2E). Our in vitro experiments were then conducted using a known hepatocytic cell line that is also amenable for mechanistic studies, in order to further examine Netrin-1 induction. We infected proliferating and dimethyl sulfoxide (DMSO)-differentiated [25] Huh7.5 cells [26] with an HCV JFH1 isolate bearing three adaptive mutations [27], or with a non-adapted genotype 1 strain [28]. Over the five- to ten-day kinetic follow-up study, HCV induced an 8-fold increase in the levels of *Netrin-1* mRNA at day eight post-infection in proliferating cultures infected with an HCV JFH1 isolate (Fig 2F) or with a genotype 1 strain (Fig 2G) and in differentiated cells (Fig 2H). These results indicate that HCV induces Netrin-1 expression in hepatocytes both in vivo and in vitro.

**Fig 3:**
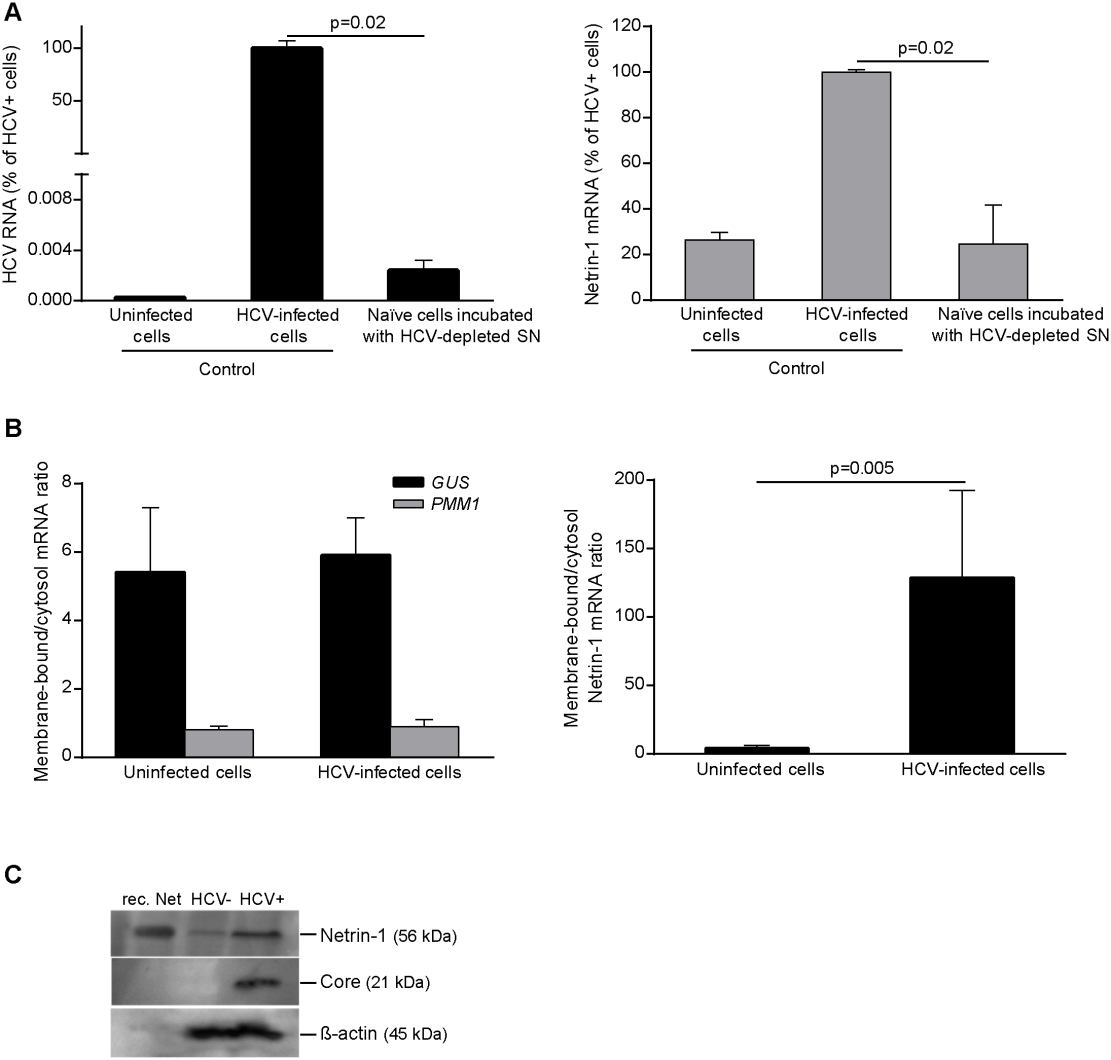
HCV increases *Netrin-1* mRNA and protein. **A**. Virions depletion experiment. Huh7.5 cells were infected by HCV over 3 d, and the supernatant (SN) was collected, depleted of HCV particles by ultracentrifugation, and added to naïve Huh7.5 cells. Levels of HCV RNA (left) and *Netrin-1* mRNA (right) were quantified by RT-qPCR. Data are represented as mean ± standard deviation (*n* = 3). **B**. HCV increases the association of *Netrin-1* mRNA with microsomes (endoplasmic reticulum [ER] membrane)-bound polysomes in Huh7.5 cells. *Netrin-1*, Glucuronidase (*GUS)*, and phosphomannomutase 1 (*PMM1)* mRNA were monitored for their partitioning in the cytosolic (free polysomes) and microsome (ER membrane-bound polysomes) compartments according to the infection status of the cells through sequential extractions followed by RT-qPCR. Statistical significance was determined using the Mann-Whitney test, *p* < 0.05. **C**. Netrin-1 protein expression is increased in HCV-positive cells. The level of Netrin-1 protein was detected in microsomes in Huh7.5 cells, naïve and HCV infected cells by immunoblotting using 200 μg of protein, anti-Netrin-1 antibody and recombinant Netrin-1 (rec. Net) as a control. Infection (core signal) and loading control (β-actin signals) are shown. The underlying data for panels in this figure can be found in S1 Data.

Next, we reasoned that substances other than virions released by HCV-infected cells might contribute to upregulating Netrin-1. To test this possibility, we incubated naïve Huh7.5 cells with conditioned medium obtained from HCV-infected Huh7.5 cultures, which had previously undergone ultracentrifugation to remove virions. The HCV-depleted conditioned medium had little effect on *Netrin-1* expression in the recipient cultures (Fig 3A), indicating that the increase in Netrin-1 expression described above was most likely mediated by the virus. Overall, these observations indicate that HCV and Netrin-1 levels are linked in individual patients, as well as across the cohort, and that HCV infection is able to induce Netrin-1 expression in vitro.

Having established that Netrin-1 expression was strongly induced by HCV, we were interested in studying the mechanisms underlying this expression. As is frequently observed in studies on Netrin-1 using cultured non-neural cell lines, Netrin-1 was difficult to detect at the protein level in Huh7.5 cells. In a novel and indirect approach to study Netrin-1 protein production, we investigated the association of *Netrin-1* mRNA with endoplasmic reticulum (ER) membrane-bound polysomes, in which the translation of this secreted protein takes place, or with free polysomes, upon infection. The partitioning of the glucuronidase (*GUS)* and phosphomannomutase 1 (*PMM1)* mRNAs, which are translated by membrane-bound and free polysomes, respectively, was also assessed as enrichment controls. Our results showed that HCV infection caused a striking enrichment in membrane-bound *Netrin-1* mRNA but did not alter the profiles of the enrichment controls (Fig 3B). Taking advantage of this subcellular fractionation approach, we submitted these previously obtained microsomes to Netrin-1 immunoblotting and observed an increase in the levels of Netrin-1 in HCV+ cells (Fig 3C). These observations indicate that HCV increases Netrin-1 translation and support the pattern of increased levels of Netrin-1 protein observed in the HCV+ clinical biopsies.

### NS5A-LARP1-Mediated Increase in Netrin-1 Production

**Fig 4:**
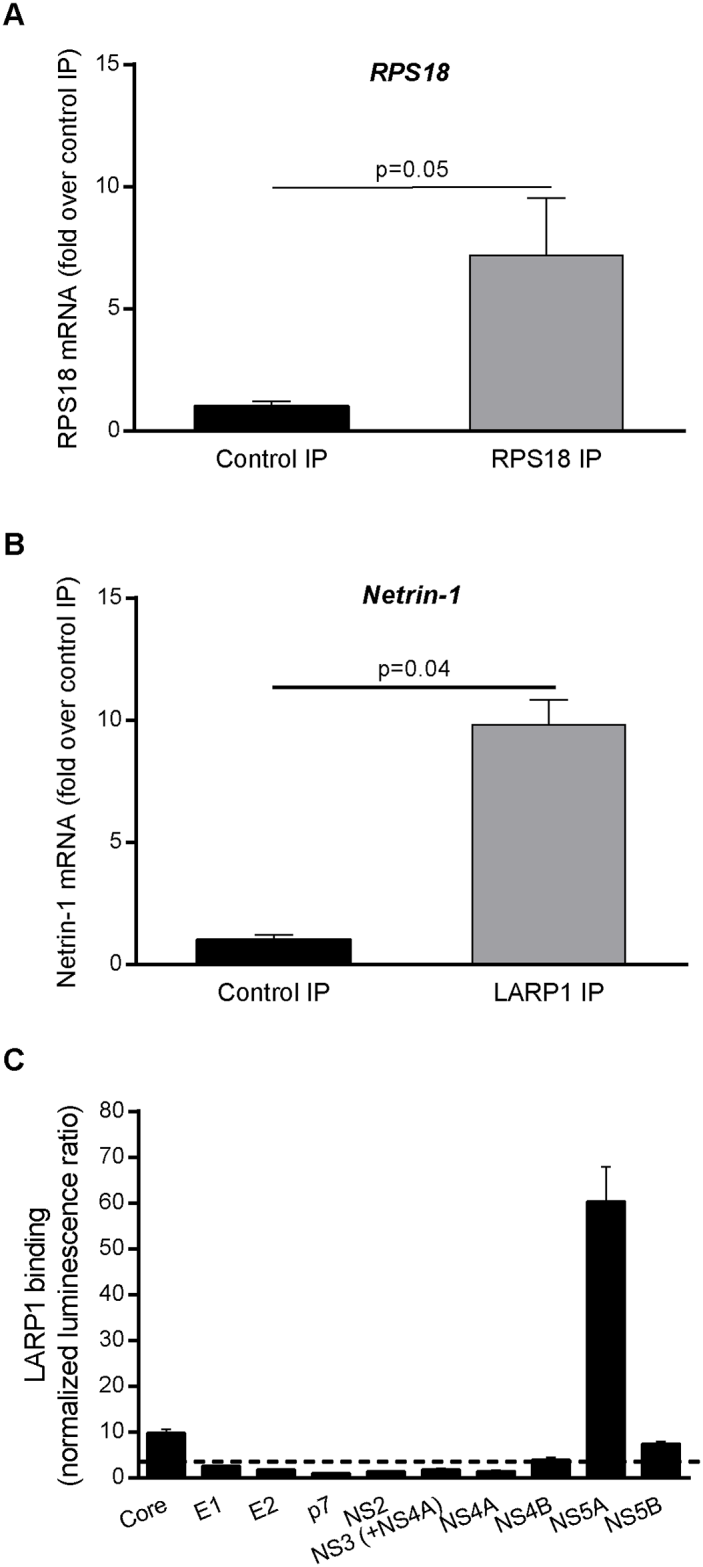
*Netrin-1* mRNA and HCV NS5A are LARP1 interactants. Quantification of ribosomal protein S18 (RPS18) (**A**) and Netrin-1 (**B**) mRNA by qPCR after immunoprecipitation using LARP1 antibody (data are represented as mean ± standard deviation, *n* = 3, Mann-Whitney test, *p* < 0.05). **C**. Huh-7.5 cells were cotransfected with a Gluc1-LARP1 plasmid and individual plasmids encoding Gluc2 fused to the indicated HCV proteins and subjected to luciferase assays. *Y*-axis represents normalized luminescence ratio (NLR). The dashed line represents background level measured using a 53-protein random reference set. Data represent means ± standard deviations (error bars) from three independent experiments in triplicates. The underlying data for panels in this figure can be found in S1 Data.

In order to investigate the HCV-related induction of the Netrin-1 protein, we examined the genetic structure of the *Netrin-1* transcript and found that its mRNA has a terminal oligopyrimidine tract (TOP) in its 5’UTR using the RegRNA2 database [29,30]. As reported previously, TOP mRNAs interact with the La-related protein 1 (LARP1; GenBank Acc. # NM_015315; Uniprot Acc. # Q6PKG0) protein during translation [31,32]. We therefore reasoned that Netrin-1 may benefit from such an interaction. Indeed, it is known that the *Netrin-1* transcript is a LARP1-binding transcript [30], and our experimental data using the LARP1-binding transcript ribosomal protein S18 (RPS18; GenBank Acc. # NM_022551) as a positive control confirmed this finding in hepatocytic cells using RNA immunoprecipitation (S2 Fig) followed by qPCR (Fig 4A and 4B).

We then searched for a specific HCV factor that could implicate LARP1 in the final Netrin-1 phenotype. We screened for potential interactions between individual HCV proteins and LARP1 using a mammalian cell-based protein-fragment complementation assay (PCA). This technique provides a highly reproducible and specific means of measuring protein interactions, including those involving membrane proteins in a cell model and in subcellular compartments [33,34]. Open reading frames (ORFs) of the ten individual HCV proteins [35] were cloned and recombined into an expression vector containing a fragment of the luciferase reporter (GLuc1-A). These plasmids were expressed in Huh7.5 cells along with a plasmid encoding for the complementary luciferase fragment (GLuc2-A) fused to LARP1. This screen revealed a novel interaction between LARP1 and HCV NS5A (Fig 4C). The apparent affinity of the NS5A-LARP1 binding was comparable to or greater than that of NS5A binding to its known partners VAPA, GRB2, RAF1, PITX1, and TP53 (reviewed in He et al. [36]). In contrast, core and NS5B exhibited weak binding close to the detection threshold, and E1, E2, P7, NS2, NS3 (expressed either individually or with Flag-tagged NS4A), NS4A, and NS4B exhibited an even lower level of binding. Interestingly, NS5A is an ER-bound protein (reviewed, for instance, in reference [36]) and is, therefore, theoretically capable of bringing its interacting partners closer to this subcellular compartment.

**Fig 5:**
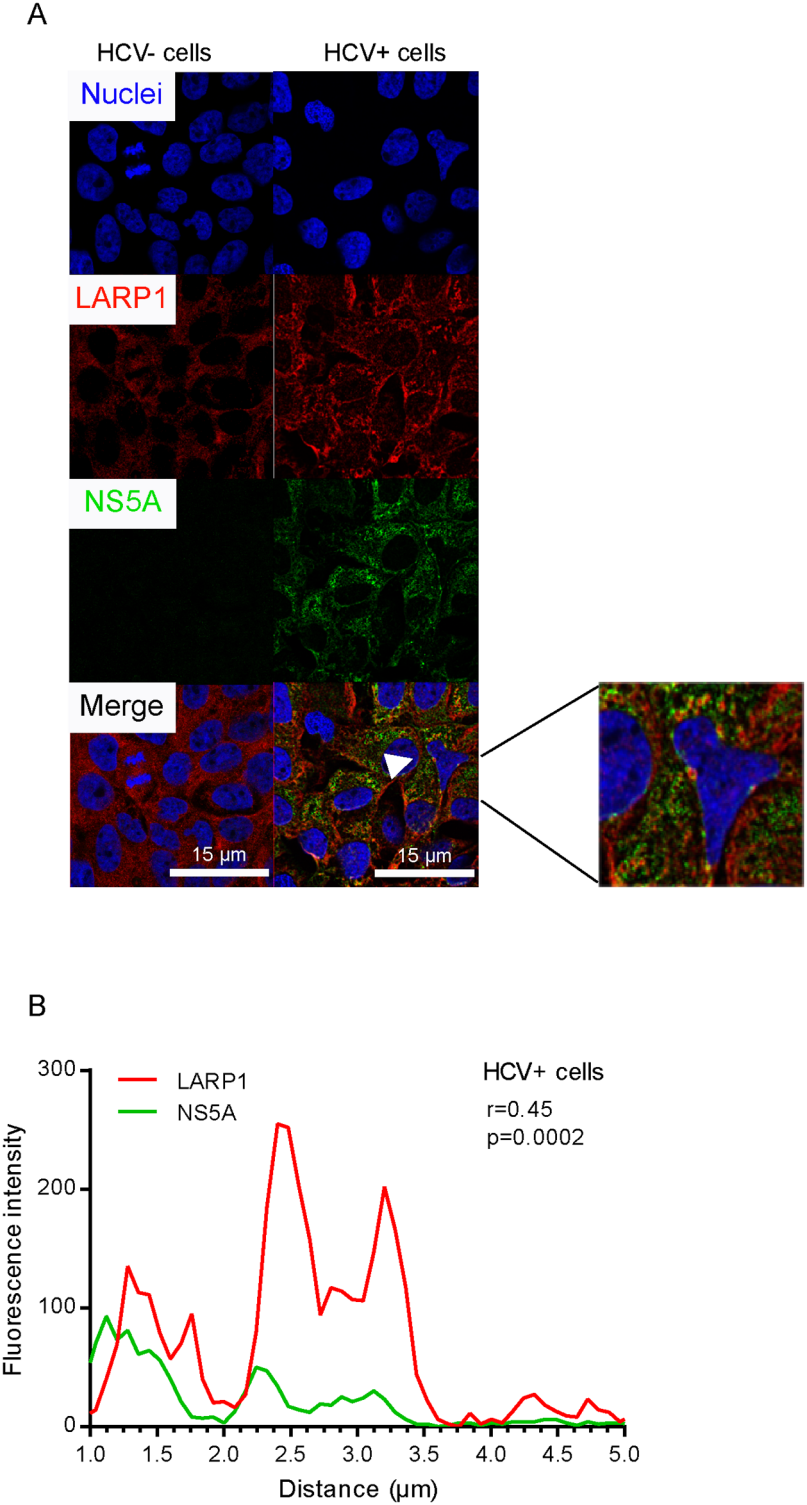
HCV NS5A colocalizes with LARP1 in infected Huh7.5 cells. **A**. Cells were infected by HCV over 4 d prior to fixation, NS5A and LARP1 double-staining, and confocal microscopy. Nuclei were counterstained with Hoechst 33342. LARP1 (red) and NS5A (green) were detected using Alexa-594 and Alexa-488, respectively. Overlays were generated by the ImageJ software. Solid arrow shows colocalization site. Bar = 15 μm. **B**. Statistical assessment of the colocalization of LARP1 and NS5A. Red (LARP1) and green (NS5A) fluorescence intensities were measured for each pixel along a 5-μm horizontal line centered around the arrow tip using the Plot Profile function of the ImageJ software. Spearman correlation coefficients for each couple of intensity values are shown. The underlying data for panels in this figure can be found in S1 Data.

**Fig 6:**
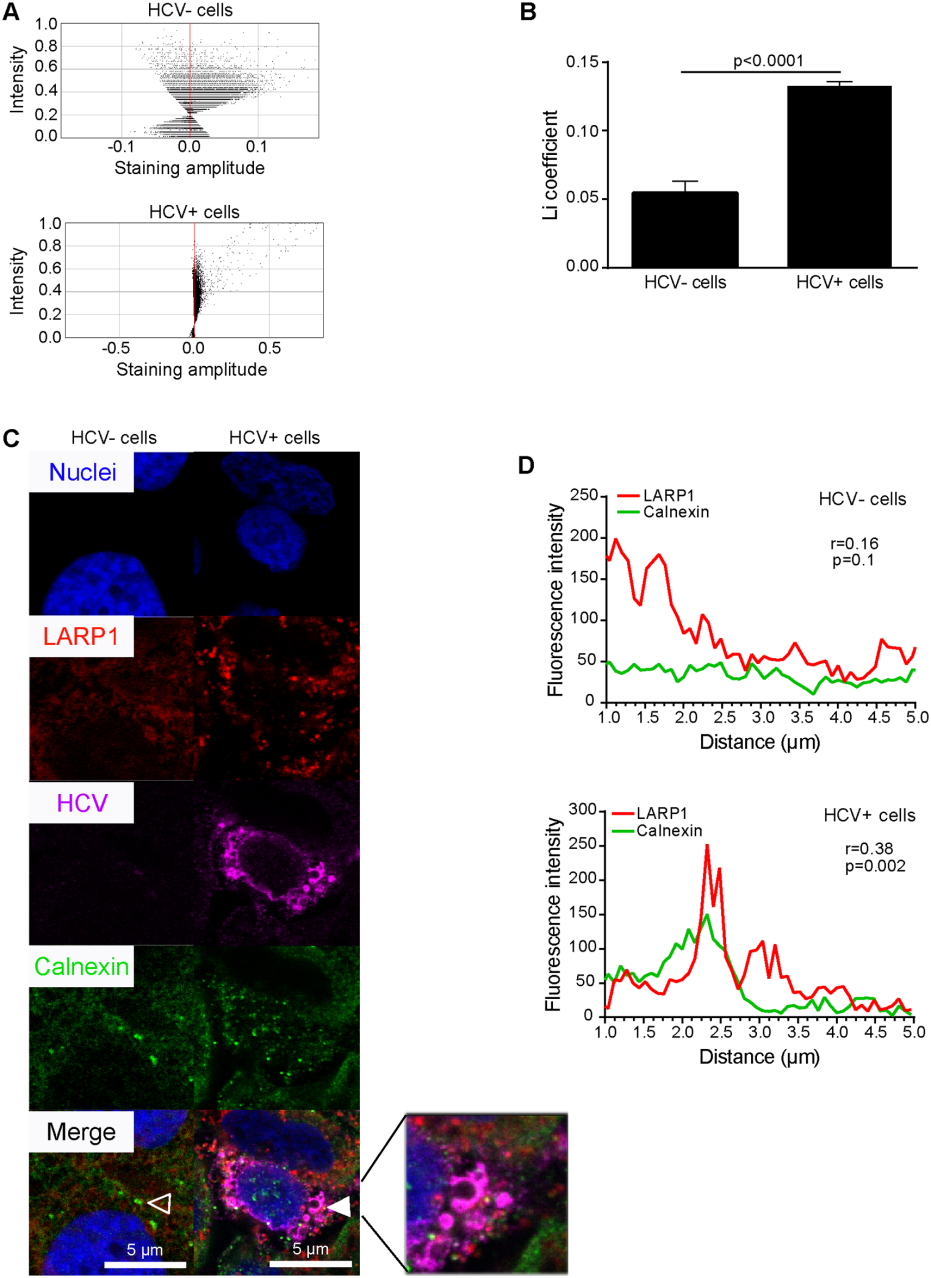
LARP1 relocalizes to the ER upon HCV infection. Cells were infected by HCV for 4 d prior to fixation, LARP1, NS5A, and calnexin staining, and confocal microscopy. **A**. Li diagrams and Li coefficient (**B**) calculations for LARP1 and Calnexin expression. Pixels present on the left and right sides of the *y*-axis, i.e., associated with negative and positive staining amplitude values, indicate exclusion and colocalization, respectively. Li coefficients were calculated using the JACop plugin from the ImageJ software (http://rsb.info.nih.gov/ij/plugins/track/jacop.html). Twelve to 15 random fields were acquired per biological sample, totaling 250–300 cells analyzed for each biological sample in a given single experiment (*n* = 3). **C**. Representative immunofluorescence-based localization of LARP1, HCV core, and calnexin. Nuclei were counterstained with Hoechst 33342. LARP1 (red), core (magenta), and calnexin (green) were detected using Alexa-617, Alexa-594, and Alexa-488, respectively. Overlays were generated by the ImageJ software. Open and solid arrows show no or total colocalization, respectively. Bar = 5 μm. **D**. Statistical assessment of the colocalization of LARP1 and Calnexin. Red (LARP1) and green (Calnexin) fluorescence intensities were measured for each pixel along a 5-μm horizontal line centered around the arrow tips in (**C**) using the Plot Profile function of the ImageJ software. Spearman correlation coefficients for each couple of intensity values are shown. The underlying data for panels in this figure can be found in S1 Data.

**Fig 7:**
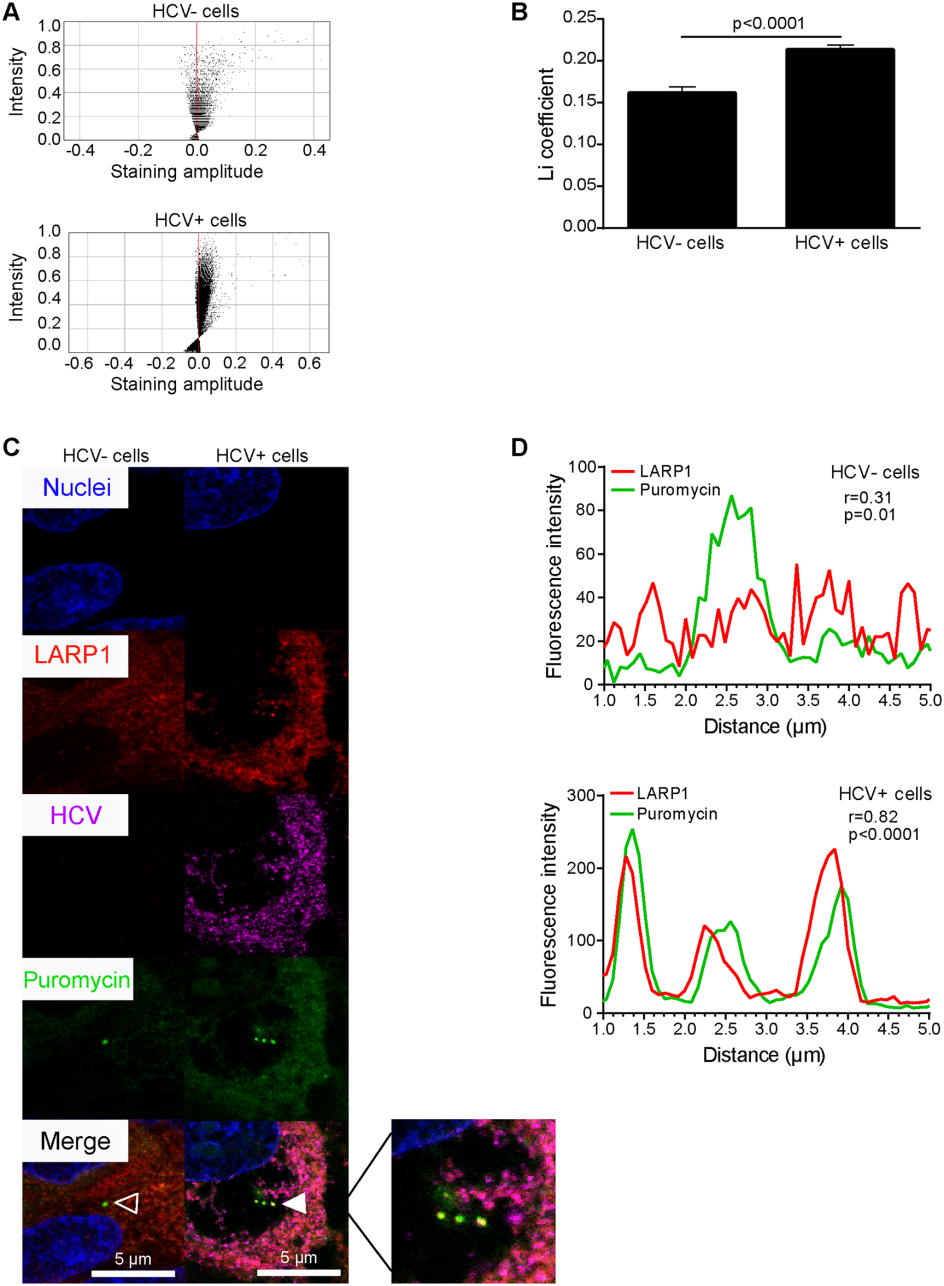
LARP1 relocalizes to active translation complexes upon HCV infection. Cells were infected by HCV over 4 d and treated with puromycin (dose) prior to fixation, LARP1, NS5A, and puromycin staining, and confocal microscopy. **A**. Li diagrams and Li coefficient (**B**) calculations for LARP1 and puromycin expression. Pixels present on the left and right sides of the *y*-axis, i.e., associated with negative and positive staining amplitude values, indicate exclusion and colocalization, respectively. Li coefficients were calculated using the JACop plugin from the ImageJ software (http://rsb.info.nih.gov/ij/plugins/track/jacop.html). Twelve to 15 random fields were acquired per biological sample, totaling 250–300 cells analyzed for each biological sample in a given single experiment (*n* = 3). **C**. Representative immunofluorescence-based localization of LARP1, NS5A, and puromycin. Nuclei were counterstained with Hoechst 33342. LARP1 (red), NS5A (magenta), and puromycin (green) were detected using Alexa-617, Alexa-594, and Alexa-488, respectively. Overlays were generated by the ImageJ software. Open and solid arrows show no or total colocalization, respectively. Bar = 5 μm. **D**. Statistical assessment of the colocalization of LARP1 and puromycin. Red (LARP1) and green (puromycin) fluorescence intensities were measured for each pixel along a 5-μm horizontal line centered around the arrow tips in (**C**) using the Plot Profile function of the ImageJ software. Spearman correlation coefficients for each couple of intensity values are shown. The underlying data for panels in this figure can be found in S1 Data.

In this context, we verified whether HCV infection induced alterations in the expression pattern of LARP1 in infected cells. Since HCV NS5A appeared to bind to LARP1 with the highest affinity, we conducted immunofluorescence assays to confirm this finding. LARP1 signals were strongly reconfigured following HCV infection, adopted a granular pattern at the expense of their initial homogenous staining profile in naïve cells, and concentrated at the vicinity of lipid droplets visible as spheric structures surrounded by LARP1 and NS5A staining (Fig 5A). We confirmed that the HCV NS5A protein colocalized with LARP1 in infected cells using a plot profile assay (Fig 5B). This was further confirmed using calnexin, an independent ER marker. Indeed, LARP1 underwent general relocalization to ER-positive sites (i.e., relevant to translation of secreted proteins) in HCV+ cells, especially at the vicinity of classically core-decorated lipid droplets (Fig 6, zoomed insert). Li colocalization parameters presented in diagram Fig 6A and the coefficient (Fig 6B) between LARP1 and calnexin were significantly upregulated (>2.5-fold) following HCV infection (for more details on Li values, see S1 Text). Representative images and plot profiles (r = 0.16; *p* = 0.1 in naïve cells versus r = 0.38; *p* = 0.002 in HCV+ cells) of these colocalization levels are shown in Fig 6C and 6D, respectively. To determine whether LARP1 was localized in translationally active sites within infected cells, we compared LARP1 aggregation sites with puromycin(+) areas using the ribopuromycylation method [37]. Accordingly, LARP1 had significantly accumulated in the cytosol of HCV+ cells (Fig 7). Li diagrams (Fig 7A) and coefficient (Fig 7B) were significantly upregulated (1.3-fold) in HCV+ cells. Corresponding representative images (Fig 7C) and plot profiles (r = 0.31 in naïve cells versus r = 0.82 in HCV+ cells, Fig 7D) are shown. These data (i) show that LARP1 strongly relocates to ER-associated translationally active sites upon HCV infection, which comprise areas adjacent to lipid droplets, and (ii) led us to investigate whether the HCV-induced increase in Netrin-1 translation was, in turn, LARP1-mediated.

**Fig 8:**
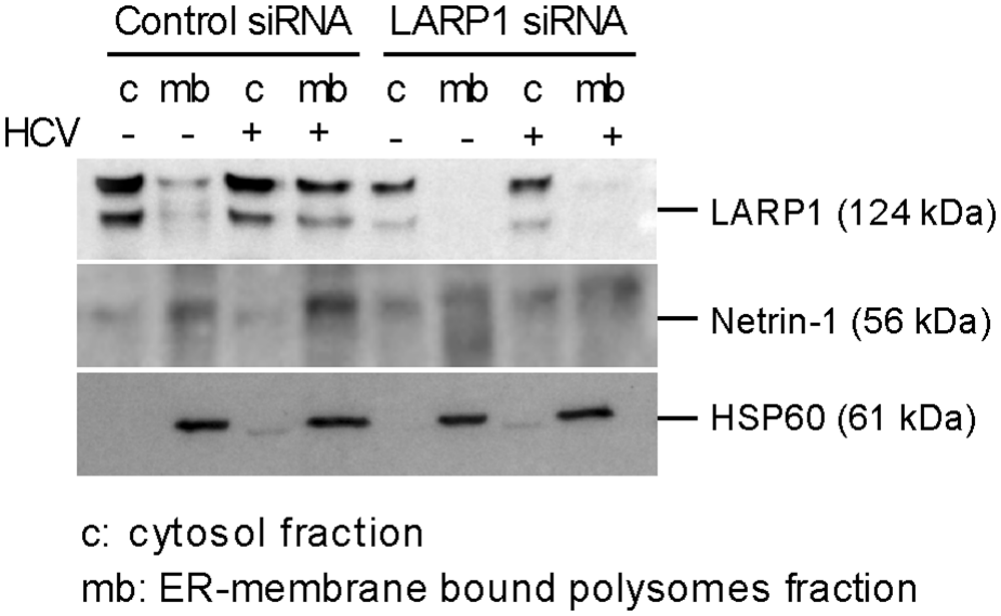
LARP1 regulates Netrin-1 microsomal translation. Huh7.5 cells were transfected with control and LARP1-specific siRNAs, and infected by HCV at MOI 0.1 over 4 d. Cells were lysed and analyzed by immunoblotting for LARP1 knockdown as shown in S3 Fig. LARP1 depletion decreases the expression of Netrin-1 protein in microsomes in Huh7.5 cells. LARP1, Netrin-1, and HSP60 proteins were monitored for their partitioning in the cytosolic (free polysomes) and microsome (ER membrane-bound polysomes) compartments, according to the infection status of the cells through sequential extractions followed by immunoblotting. HCV core was used as an infection control.

LARP1 expression is conditioned by NS5A in infected cells. We reasoned that the siRNA-based modulation of LARP1 expression in HCV+ cells should alter the microsomal levels of Netrin-1 in these cells. To test this hypothesis, we first assessed LARP1 knockdown by immunoblotting (S3 Fig) and subsequently separated the microsomes from the cytosol by performing subcellular fractionation with the HSP60 marker. We then evaluated Netrin-1 levels in both types of samples after modulating the expression of LARP1. In agreement with previous immunofluorescence data, levels of LARP1 and Netrin-1 increased in the microsomal compartment upon HCV infection, while Netrin-1 decreased in this subcellular fraction upon depletion of LARP1 (Fig 8). Therefore, the virus NS5A-mediated relocalization of LARP1 toward ER-associated translationally active sites accounts for the HCV-related increase in Netrin-1. Consistently with the secreted protein status of Netrin-1, this HCV-related increase occurs primarily in the secretory, microsomal machinery of the cell.

### Netrin-1 Exerts a Pro-Viral Effect by Enhancing the Infectivity of HCV Particles via Its Inhibition of the UNC5A Receptor

**Fig 9:**
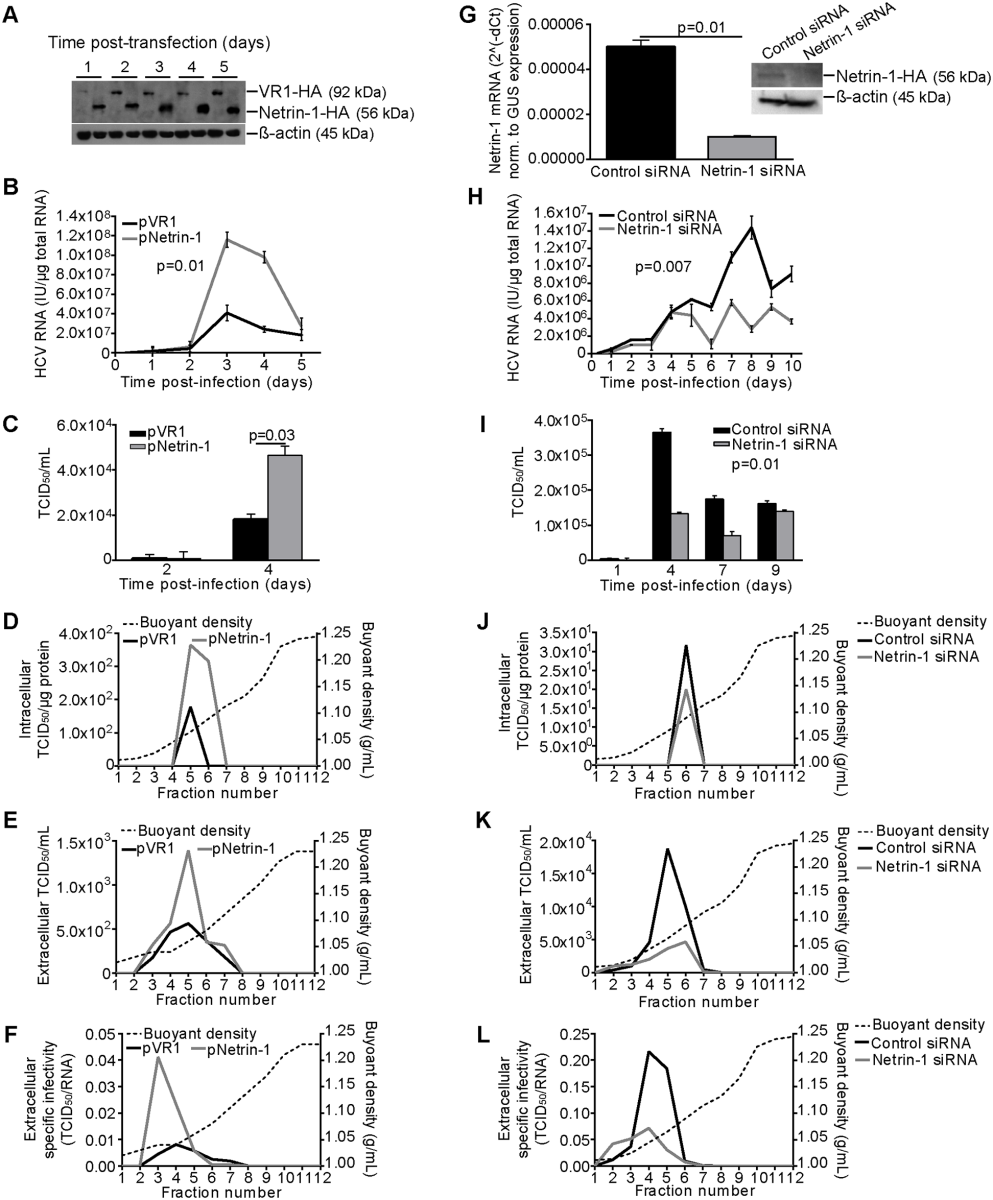
Netrin-1 overexpression increases HCV RNA and specific infectivity of HCV virions in vitro whereas Netrin-1 depletion decreases HCV RNA and specific infectivity in vitro. **A.** Detection of VR1-HA and Netrin-1-HA in transfected Huh7.5 cells by immunoblotting with the anti-HA antibody. **B**. Netrin-1 overexpression enhances intracellular HCV RNA. Huh7.5 cells were transfected with the VR1-HA or the Netrin-1-HA plasmid and infected at a MOI of 0.1 the day after seeding. Intracellular HCV RNA was quantified by RT-qPCR at each time point (data are shown as mean ± standard deviation, *n* = 3, Wilcoxon test, *p* < 0.05). **C.** Supernatant infectivity was quantified on days two and four post-infection by the TCID_50_ method (*n* = 3, Mann-Whitney test, *p* < 0.05.). **D.** Intracellular infectivity was quantified on day four post-infection by the TCID_50_ method (*n* = 3). **E**. Density of infectivity values was quantified for each collected sucrose gradient fraction, 3 d post-infection, by the TCID_50_ method (*n* = 3). Buoyant densities were determined by refractometry. **F**. Netrin-1 increases the specific infectivity of virions. Specific infectivity was calculated for each collected fraction using the TCID_50_/HCV RNA ratio and refractometry (*n* = 3). **G.** Assessment of *Netrin-1* mRNA knockdown. Cells were initially transfected with control and Netrin-1-specific siRNAs, then harvested at the indicated time points and processed for *Netrin-1* mRNA quantification by RT-qPCR or by immunoblotting in microsomes using an anti-Netrin-1 antibody (data are shown as mean ± standard deviation, *n* = 3, Mann-Whitney test, *p* < 0.05). **H**. Netrin-1 depletion impedes HCV. Huh7.5 cells were transfected with siRNAs, infected at a MOI of 0.1 24 h after seeding, and trypsinized at day five post-infection before a second siRNA transfection. Intracellular HCV RNA was quantified by RT-qPCR at each time point (data shown as mean ± standard deviation, *n* = 3, Wilcoxon test, *p* < 0.05). **I**. Supernatant infectivity was quantified on days one, four, seven, and nine post-infection by the TCID_50_ method (*n* = 3, Wilcoxon test, *p* < 0.05). **J**. Intracellular infectivity was quantified on day four post-infection by the TCID_50_ method (*n* = 3). **K**. 6 d post-infection, culture supernatants were subjected to sucrose gradient centrifugation, and infectivity was quantified in each collected fraction by the TCID_50_ method. Buoyant densities were determined by refractometry (*n* = 3). **L**. Netrin-1 depletion decreases specific infectivity of virions. Specific infectivity was calculated for each collected fraction using the TCID_50_/HCV RNA ratio. (*n* = 3). The underlying data for panels in this figure can be found in S1 Data.

These initial findings prompted us to investigate whether Netrin-1 produced by cultured cells was, in turn, able to promote HCV replication and/or propagation. HCV-infected proliferative Huh7.5 cells were transfected with a Netrin-1 expression plasmid. Intracellular HCV RNA, viral RNA release, supernatant infectivity, and virion-specific infectivity were then monitored. The expression vector produced a modified Netrin-1 containing the hemagglutinin (HA) epitope at its carboxyl-terminus, while a plasmid expressing HA-tagged vanilloid receptor (VR1-HA) served as a control. Immunoblotting analyses confirmed expression of both proteins in the transfected cells (Fig 9A). Plasmid-delivered Netrin-1-HA produced a 3-fold increase in the intracellular HCV RNA compared to cells transfected with VR1-HA (Fig 9B) and was accompanied by a 2.5-fold increase in supernatant infectivity, measured using the TCID_50_ protocol (Fig 9C). Furthermore, overexpression of Netrin-1 resulted in a 2-fold increase in the level of intracellular infectivity (Fig 9D) and likewise increased the infectivity peak of the released virions (Fig 9E). Since LARP1 intriguingly concentrates at the ER and in the proximity of lipid droplets, an important viral budding site [38], upon infection, we tested whether accumulation of Netrin-1 in microsomes could foster increased morphogenesis, through the evaluation of the specific infectivity of virions. This viral parameter is defined by the ratio of biological infectivity values, expressed as TCID_50_ units, and viral RNA copy numbers of the sample. To achieve this, we plotted TCID_50_/extracellular HCV RNA ratios against their buoyant densities for Netrin-1- and control VR1-transfected samples. Netrin-1 overexpression caused a 4-fold increase in the specific infectivity of released virions (Fig 9F), suggesting that the protein induces alterations in the virus particle. Netrin-1 also resulted in a viral increase of the poorly infectious, high-density fractions (S4 Fig).

The DR hypothesis states that, ligand withdrawal induces receptor activation for subsequent death signaling. As Netrin-1 is known to promote cell survival [39], we performed a set of experiments resulting in the overexpression or depletion of Netrin-1, to test whether its effect on HCV occurs via a cell death protection-dependent mechanism. We initially performed cell death-related assays on Huh7.5 cells during the entire course of the transfection experiments, and observed that neither caspase-3 activity nor cell proliferation (S5 Fig), known to be beneficial for HCV replication in vitro, were altered by the forced expression of Netrin-1. This suggests that Netrin-1 does not exert its proviral effect through the death-related DR function of its cognate receptors

We then incubated recombinant soluble Netrin-1-Fc on HCV in Huh7.5 cells. In both cell systems, Netrin-1-Fc induced a significant (up to 2-fold) increase in the level of intracellular HCV RNA. TCID_50_ assays showed that while extracellular release of HCV RNA was not changed, it produced a 2-fold increase in the level of infectivity of the supernatant (S6 Fig). Netrin-1-Fc did not affect caspase-3 activity in Huh7.5 cells. Results of neutral red assays indicated that Netrin-1-Fc did not influence the viability of the Huh7.5 cells over time regardless of their HCV infection status (S7 Fig). Similar results were obtained when using a distinct recombinant soluble Netrin-1, Netrin-1-FLAG (S8 Fig), strengthening our previous findings that Netrin-1 does not promote HCV infection by protecting against cell death.

In turn, we also studied the effect of Netrin-1 depletion on all previously depicted viral parameters. The efficiency of the Netrin-1 siRNA was assessed by qPCR and immunoblotting (Fig 9G). SiRNA-mediated Netrin-1 knockdown was associated with a 3-to-4-fold decrease in the level of intracellular HCV RNA (Fig 9H; see also SI3 for dose-dependence data) and caused a 2-fold decrease in the level of global infectivity of the HCV supernatant (Fig 9I), as well as a decrease in intracellular infectivity (Fig 9J). Infectivity of the released virions was also clearly impaired by Netrin-1 depletion (Fig 9K). Consistently with results generated upon overexpression, Netrin-1-silencing caused a 3-fold decrease in the specific infectivity levels of released virions (Fig 9L), another indication that Netrin-1 induces alterations in the virus particle that are virion-density unrelated (S9 Fig). Finally, Netrin-1-depleted cells were also analyzed for caspase-3 activity and cell viability to rule out the possibility that the positive effects of Netrin-1 on HCV infection were due to protection against cell death. No effect of Netrin-1 depletion on caspase-3 activity (S10A Fig) or cell viability (S10B Fig) was observed. Importantly, in an approach to deplete Netrin-1 in an RNAi-independent fashion, infected Huh7.5 cells were exposed to a recombinant anti-Netrin-1 monoclonal antibody. This treatment resulted in a 3-fold decrease in intracellular HCV RNA (S11A Fig) and a 5-fold decrease in supernatant infectivity (S11B Fig).

**Fig 10:**
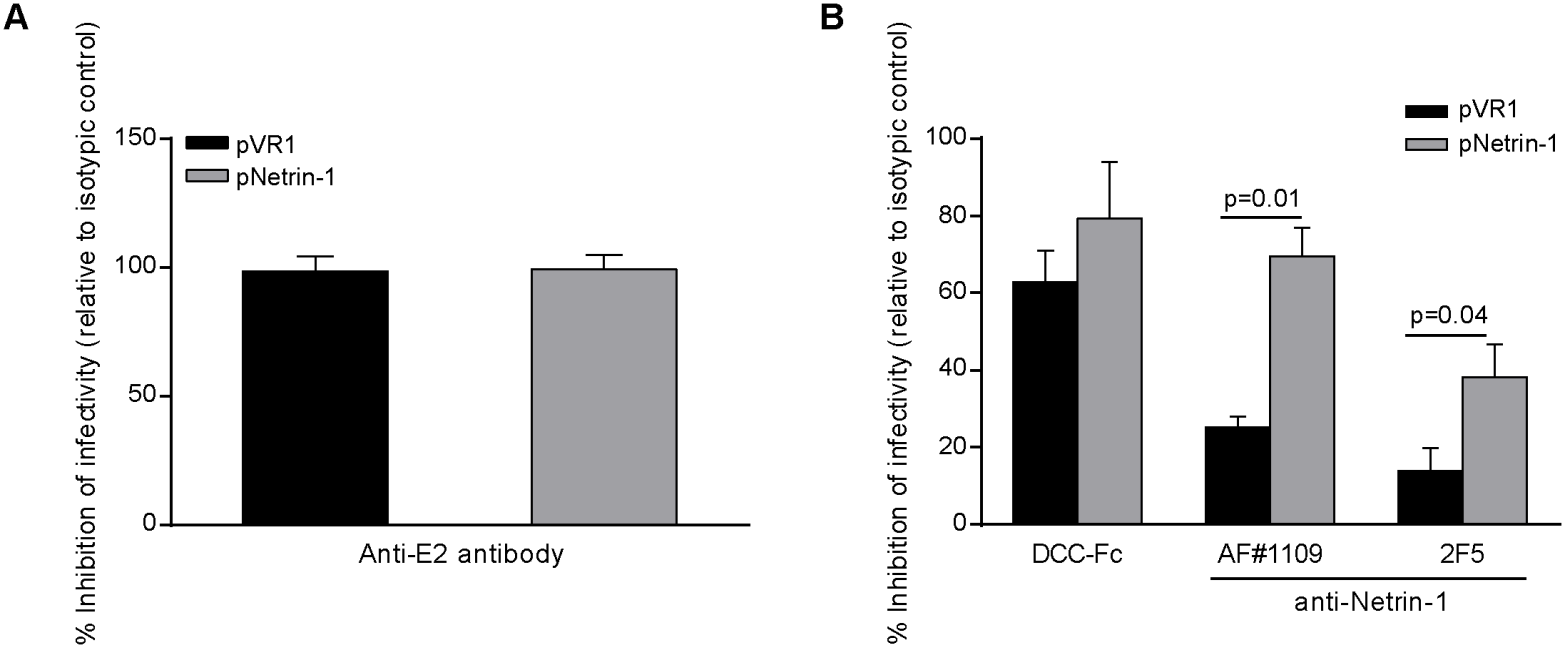
Netrin-1 is virion-bound and participates in the infectivity of viral particles. HCV particles produced in cells overexpressing Netrin-1 have been preincubated with Netrin-1 antagonists and TCID_50_ has been performed. **A**. Method validation: inhibition of HCV infectivity after incubation with anti-E2 antibody. The anti-E2 antibody was used as a control of neutralization of infectivity. **B**. Inhibition of HCV infectivity after incubation with three distinct Netrin-1 antagonists (data are represented as mean ± standard deviation, *n* = 3, Mann-Whitney test, *p* < 0.05). The underlying data for panels in this figure can be found in S1 Data.

Having shown that the modulation of Netrin-1 was capable of influencing specific infectivity levels of the HCV virions, in a context devoid of alterations in cell integrity, we investigated whether Netrin-1 could potentially be a component of the particles, since it also concentrates in the ER. We performed neutralization assays followed by TCID_50_ quantification. Anti-E2-based neutralization (versus its RO4 isotype) [23] served as a positive control (Fig 10A), while a recombinant form of the DCC (deleted in colorectal cancer) receptor of Netrin-1 (compared with the same heat-inactivated receptor) and two distinct anti-Netrin-1 antibodies (compared with their respective isotypic IgG controls) were used to evaluate the effect of virus production on Huh7.5 cells (Fig 10B). Neutralization decreased the initial level of infectivity by up to 80%, and this inhibition was enhanced by Netrin-1 overexpression in the initial virus-producing cells, suggesting that Netrin-1 participates in HCV infectivity as a candidate part of the viral particle. The effects of Netrin-1 were also tested in Huh7-derived cell lines containing subgenomic HCV replicons [40], a system that does not generate viral particles. Results showed that Netrin-1 did not alter levels of HCV RNA (S12 Fig). These observations confirm that the increase in HCV levels mediated by Netrin-1 occurs at the level of the assembly/morphogenesis, with a specific impact on the level of infectivity of the virions, but show no effect on viral RNA replication.

Netrin-1 exerts most of its known activities by interacting with the DRs DCC and UNC5 [41,42]. In order to identify the receptor transducing the pro-HCV activity of Netrin-1, we quantified the levels of expression of *UNC5s* and *DCC* in Huh7.5 and PHH cells, as well as in tissue biopsies. *UNC5A* (GenBank Acc. # NM_133369), *B* (GenBank Acc. # NM_170744), and *D* (GenBank Acc. # NM_080872) transcripts were readily detectable in Huh7.5 cells, while *UNC5C* (GenBank Acc. # NM_003728) levels remained marginal and *DCC* (GenBank Acc. # NM_005215) mRNA was neither expressed in Huh7.5 cells nor in PHH. *UNC5* profiles were similar in Huh7.5 cells, PHH, and liver tissues (S13 Fig), suggesting that the in vitro setting presented in this study was a representative model for the UNC5 DR profile in patients.

**Fig 11:**
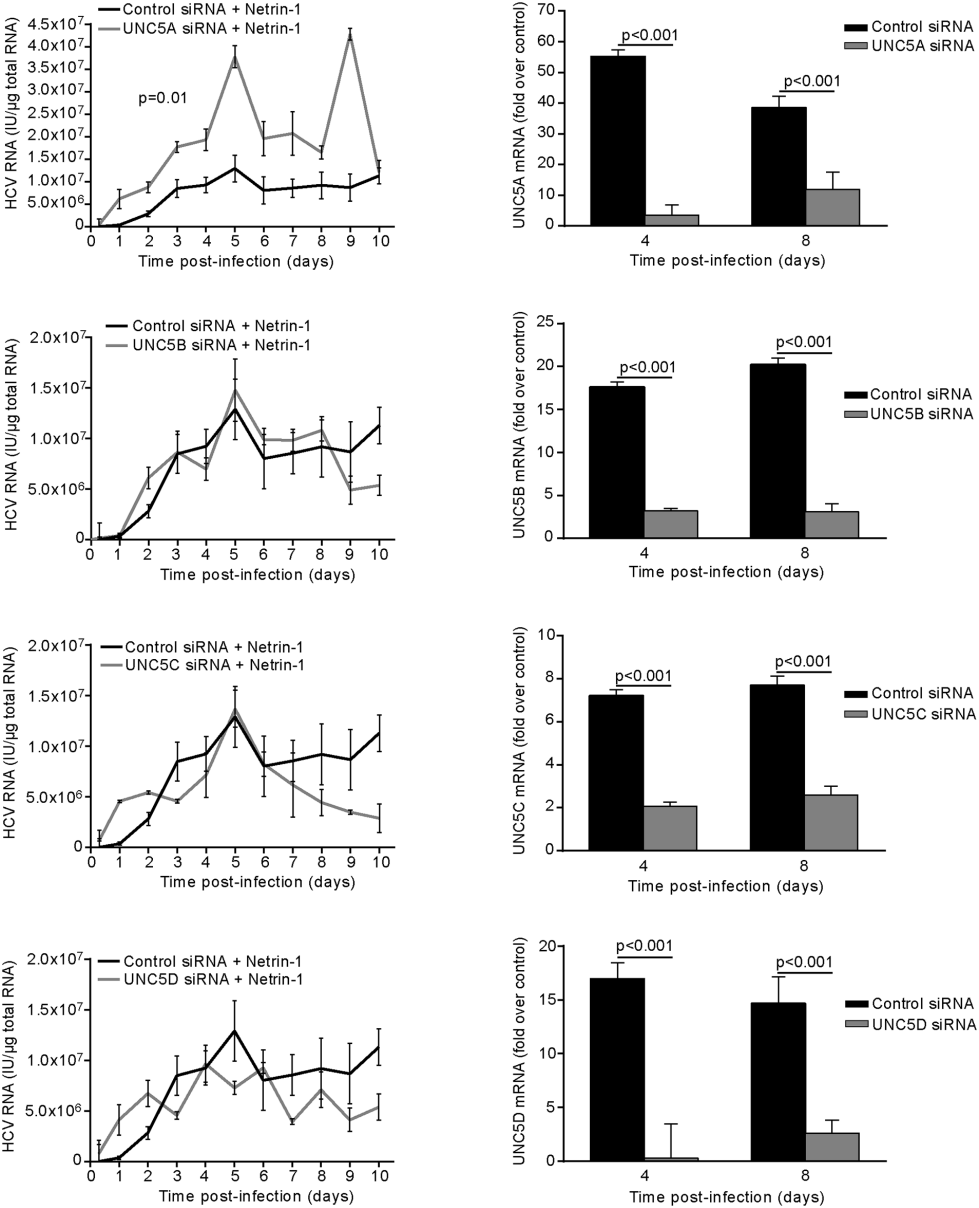
Netrin-1 increases HCV through the UNC5A receptor. Huh7.5 cells were transfected with siRNA against each UNC5 receptor or with a nontargeting control siRNA and infected at a MOI of 0.1 24 h after seeding. Cells were then trypsinized at day five post-infection before undergoing a second siRNA transfection. Recombinant soluble Netrin-1-Fc was added to the medium 12 h after transfection. Intracellular HCV RNA was quantified by RT-qPCR at each time point (left-hand graphs; data are represented as mean ± standard deviation, *n* = 3, Wilcoxon test, *p* < 0.05), while siRNA-mediated knockdown was quantified for each *UNC5* transcript on days four and eight post-infection by RT-qPCR (right-hand graphs; data shown as mean ± standard deviation, *n* = 3). The underlying data for panels in this figure can be found in S1 Data.

Based on these results, we set out to identify which of the UNC5 receptors was responsible for mediating the effects of Netrin-1 by monitoring HCV in Netrin-1-Fc-treated Huh7.5 cells, which had previously been subjected to siRNA-mediated depletion of each individual *UNC5* transcript. Depletion of UNC5A alone induced an up to 4-fold increase in the levels of HCV (Fig 11, left column). RT-qPCR conducted to detect the *UNC5* transcripts confirmed the efficacy of the siRNAs (Fig 11, right column). These results were subsequently validated using RNAi-based depletion and plasmid-mediated forced expression approaches of UNC5A (Uniprot Acc. # Q6ZN44) on viral parameters. Indeed, intra/extracellular infectivity parameters increased and decreased by 3-fold to 6-fold upon UNC5A depletion or overexpression, respectively (S14 Fig). These results demonstrate that Netrin-1 exerts its pro-HCV effect via inhibition of the UNC5A receptor that itself decreases Netrin-1’s proviral effect. They also indicate that UNC5A-related functions ultimately condition infectivity of the virus particle, through increased viral propagation inducing enhanced Netrin-1 expression.

### Netrin-1 Modulates HCV Entry through the EGFR

The EGFR (GenBank Acc. # K03193; Uniprot Acc. # P00533) is a host receptor necessary for HCV entry [3,4,43] that acts by promoting the formation of the CD81-CLDN1 viral capture complex at the level of the membrane [3]. Since the altered signaling of both Netrin-1 and EGFR plays a widespread role in cancer, we examined whether the two proteins might be functionally connected.

In order to study the response of EGFR concomitantly to Netrin-1 modulation, our experiments were conducted in EGF stimulation synchronized settings. In this context, siRNA-mediated knockdown of Netrin-1 induced a decrease in the level of EGFR at the plasma membrane level, while forced expression of Netrin-1 resulted in an increase in EGFR levels (S15A Fig). Netrin-1 had no effect on the levels of plasma membrane-associated CD81 (Uniprot Acc. # P60033), which is one of the co-receptors of HCV (S15B Fig). Working in similar conditions, we then investigated whether the level of EGFR activation was sensitive to Netrin-1 fluctuations. While knockdown of Netrin-1 caused a decrease in the activation of EGFR, as measured by immunoblotting using an anti-phospho1068 antibody, forced expression of Netrin-1 increased EGFR phosphorylation (S15C Fig), an event necessary for HCV entry [43]. These data were also confirmed in serum-containing conditions (S16 Fig) and indicate that Netrin-1 participates in EGFR activation.

**Fig 12:**
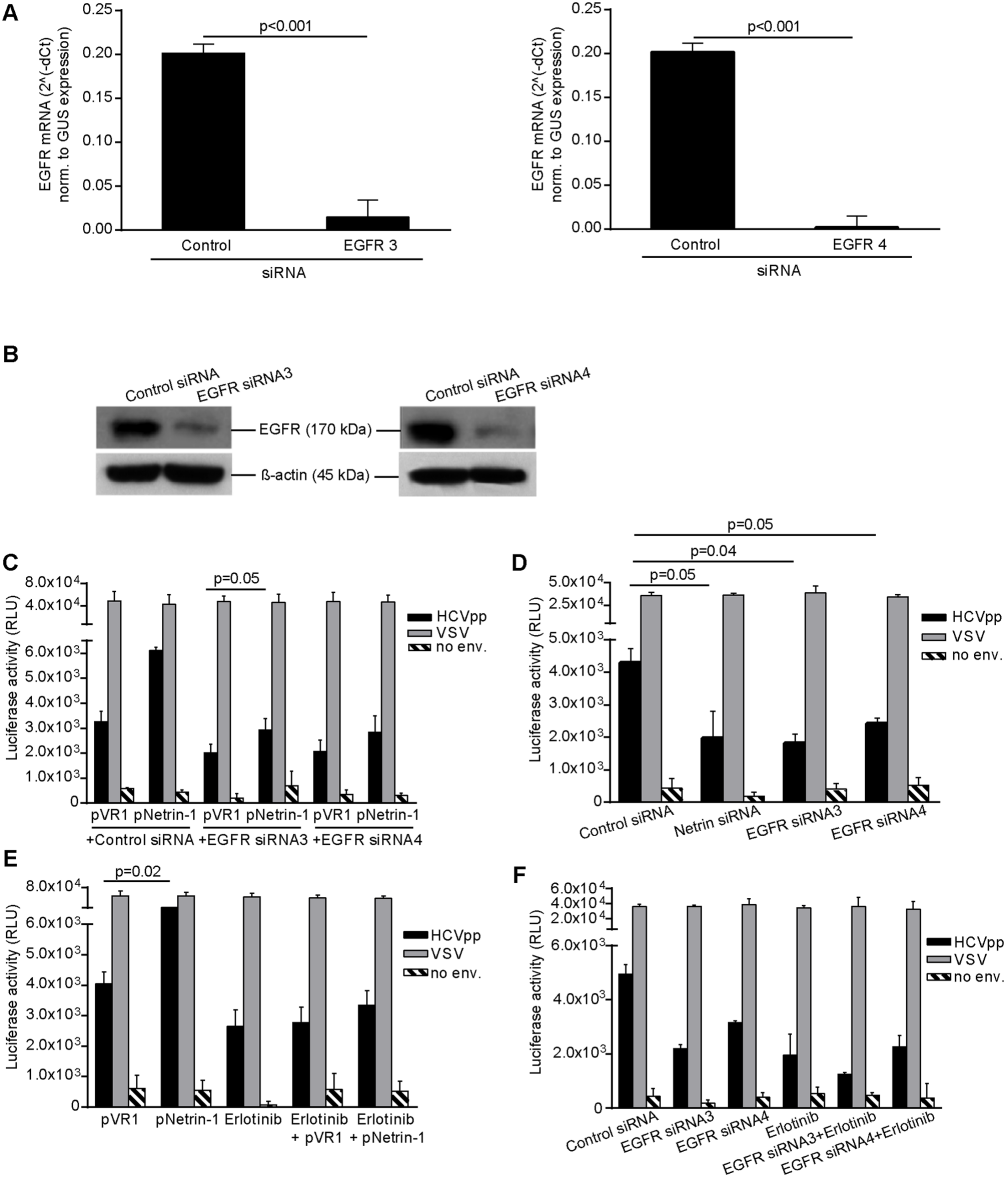
Netrin-1 enhances HCVpp entry. RNAi-mediated knockdown of EGFR. Huh7.5 cells were transfected with anti-EGFR siRNAs #3 and #4 of a previously described study [3] and infected at a MOI of 0.1. Cells were collected 3 d post-infection and analyzed by RT-qPCR (**A**) and immunoblotting using an anti-EGFR antibody targeting an extracellular epitope of the protein (**B**) (data are represented as mean ± standard deviation, *n* = 4, Mann-Whitney test, *p* < 0.05 for RT-qPCR). Parental Huh7.5 cells transfected with Netrin-1 siRNAs or plasmids or EGFR #3 or EGFR #4 siRNAs were transduced with lentiviral vectors producing HCV pseudoparticles (HCVpp), VSV-G, and envelope-negative (no env.) particles carrying a luciferase expression cassette. **C**. Effect of Netrin-1 overexpression on HCVpp entry and its reversion by EGFR depletion. **D**. Effect of Netrin-1 knockdown on HCVpp entry. **E**. Effect of Netrin-1 overexpression on HCVpp entry and its reversion by erlotinib. **F**. Effect of erlotinib treatment combined with EGFR knockdown on HCVpp entry. Cells were lysed 3 d post-infection and luciferase activity was subsequently monitored by a luminometer (data shown as mean ± standard deviation, *n* = 3, Mann-Whitney test, *p* < 0.05). The underlying data for panels in this figure can be found in S1 Data.

In order to provide a dataset at the functional level regarding the effect of Netrin-1 on the entry of the HCV and the possible role of the EGFR in this process [4], we used the HCV pseudoparticles (HCVpp) system, a pseudotyped HCV glycoprotein-expressing lentiviral tool, widely used for entry quantification assays in hepatitis C research [44]. Experiments were carried out in cells transfected with the Netrin-1 expression plasmid and/or the EGFR siRNA, while cells transfected with the VR1 expression plasmid served as negative controls. SiRNA-mediated EGFR knockdown was tested at the mRNA and protein levels (Fig 12A and 12B). Forced expression of Netrin-1 caused a 2-fold increase in the entry of HCVpp, whereas values were reversed following depletion of the EGFR (Fig 12C). Netrin-1-depletion led to a 3-fold to 4-fold decrease in the entry of HCVpp, a level of inhibition comparable to that observed when cells were transfected with EGFR siRNA (Fig 12D). The entry of positive control lentivirus pseudotyped with VSV-G was insensitive to either treatment. Entry of HCVpp devoid of envelope glycoproteins served as a negative control (Fig 12C–12F). Treatment of cells with the EGFR inhibitor erlotinib interfered with their entry, similarly to interferences observed upon overexpression of Netrin-1, but did not affect the entry of particles carrying the VSV-G phenotype (Fig 12E). Erlotinib cooperated with siRNAs directed against EGFR for further entry inhibition, as previously shown (Fig 12F) [4]. These data support the hypothesis that Netrin-1 heightens the infectivity of an inoculum by increasing the susceptibility of target cells to the entry of HCV.

Immunoblotting experiments conducted to detect the EGFR protein in total cell lysates showed that silencing or overexpression of Netrin-1 did not alter protein levels (S17 Fig). Since, in contrast, Netrin-1 was found to modulate the EGFR at the level of the plasma membrane (S15 and S16 Figs), we verified whether Netrin-1 was able to modulate its recycling upon experimental binding with its cognate ligand EGF. RT-qPCR and immunoblotting results revealed that stimulation of Netrin-1-silenced or Netrin-1-overexpressing Huh7.5 cells with EGF, for 5 or 15 min, affected neither EGFR mRNA nor protein levels (S17A and S17B Fig, respectively). In contrast, results of flow cytometry confirmed the positive effect of Netrin-1 on the levels of cell surface-exposed EGFR (S17C Fig, top panel), while EGF was used as an internalization control (S17C Fig, center and bottom panels). These results indicate that the EGFR is functional in the experimental setting used in the present study, and also that its global expression level is not regulated by Netrin-1.

**Fig 13:**
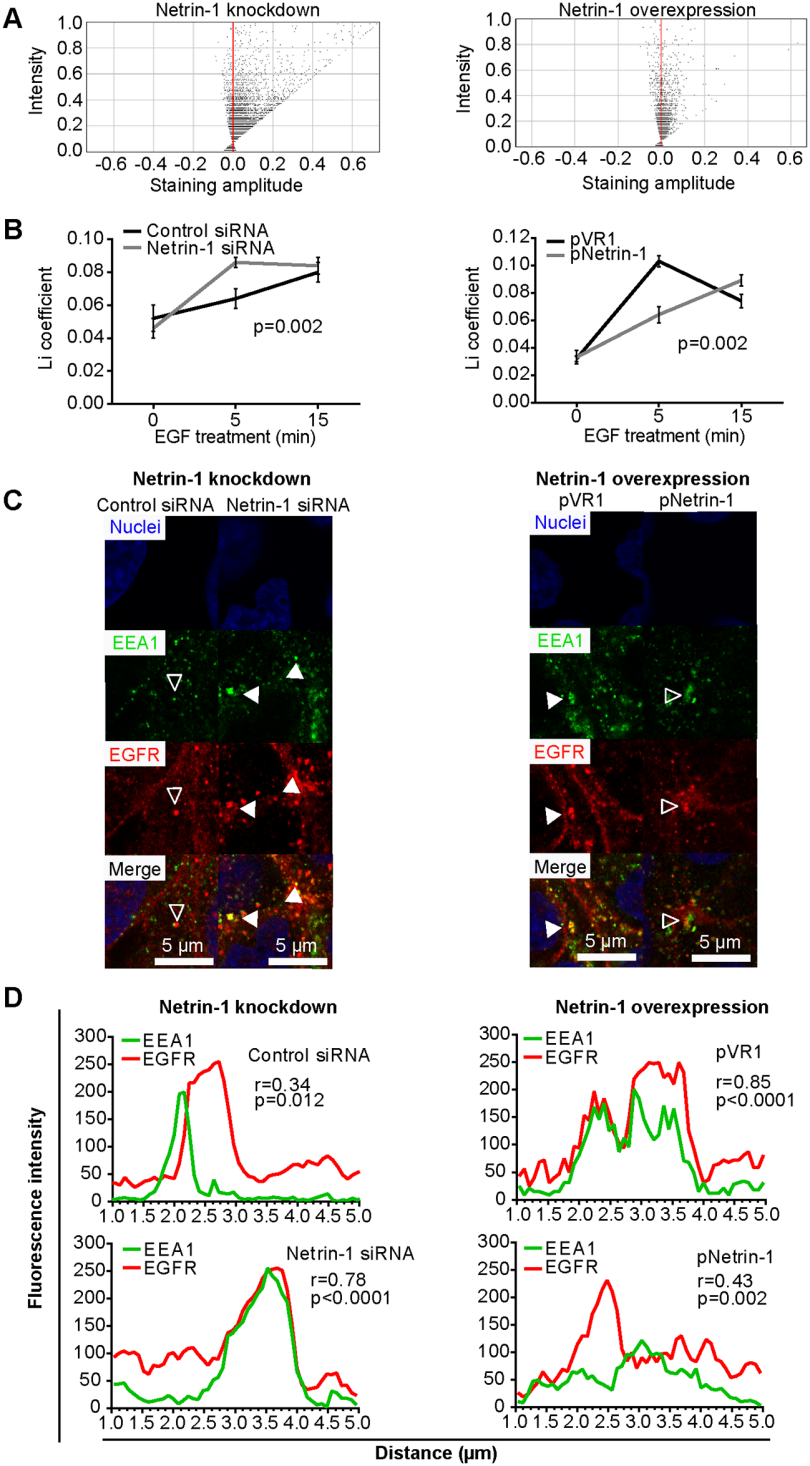
Netrin-1 impedes EGFR recycling. Huh7.5 cells infected with HCV (day four p.i.) were transfected with siRNA (control or Netrin-1-specific) or with plasmids (VR1- or Netrin-1-expressing), serum-starved for 16 h, and incubated with EGF for 5 or 15 min prior to fixation. Cells were subsequently stained for EEA1 and EGFR and visualized by confocal microscopy. **A**. Li diagrams and Li coefficient calculations (**B**) for Netrin-1 knockdown and forced expression experiments. Pixels present on the left and right sides of the *y*-axis, i.e., associated with negative and positive staining amplitude values, indicate exclusion and colocalization, respectively. Li coefficients were calculated using the JACop plugin from the imageJ software (http://rsb.info.nih.gov/ij/plugins/track/jacop.html). Twelve to 15 random fields were acquired per biological sample, totaling 250–300 cells analyzed for each biological sample in a given experiment (*n* = 2). **C**. Representative immunofluorescence-based localization of EEA1 and EGFR. Nuclei were counterstained with Hoechst 33342. EEA1 (green) and EGFR (red) were detected using Alexa-488 and Alexa-594, respectively. Overlays were generated by the Leica LAS AF software upon image acquisition. Open and solid arrows show partial and total colocalization, respectively. Bar = 5 μm. **D**. Statistical assessment of the colocalization of EEA1 and EGFR. Red (EGFR) and green (EEA1) fluorescence intensities were measured for each pixel along a 5 μm horizontal line centered around the arrow tips in (**C**) using the Plot Profile function of the ImageJ software. Spearman correlation coefficients for each couple of intensity values are shown. The underlying data for panels in this figure can be found in S1 Data.

We then investigated the interplay between HCV infection and Netrin-1 on the internalization of EGFR. HCV-infected cells were transfected with anti-Netrin-1 (siRNA) or a Netrin-1 expressing plasmid. Cells were subsequently serum-starved and incubated with EGF prior to their fixation and indirect immunofluorescence analysis to detect EGFR along with the endosomal marker EEA1 (Uniprot Acc. # Q15075). The EGFR and EEA1 signals were assessed by intensity correlation coefficient-based (ICCB) analyses (Fig 13A), based on the Li coefficient (also see detailed explanation in S3 Supporting Information) [45]. Our results reveal a strong colocalization of EGFR and EEA1 in Netrin-1 depleted cells (i.e., almost all of the pixels have positive staining amplitude values) and inversely display low levels of colocalization in cells overexpressing Netrin-1. The effect of EGF treatment on the internalization of the EGFR in Netrin-1 expression-modulated cells was then assessed by comparing changes in the Li coefficient, which is a measure of the transfer of EGFR to the early endosome. Treatment with EGF, for 5 or 15 min, resulted in an increase in the Li coefficient in the transfected cells. At the 5-min time point, the Li coefficient increased by 35% in cells depleted of Netrin-1, whereas it showed a 39% decrease in cells overexpressing Netrin-1 (Fig 13B). We showed a partial (empty arrows) and total (solid arrows) colocalization of EGFR and EEA1 (Fig 13C) by immunofluorescence in Netrin-1 overexpressing and depleted cells, respectively. The plot profiles of the fluorescence intensities of EEA1 and EGFR signals were measured across a five-micron line located at the tip of each arrowhead. Spearman correlation coefficients between EEA1 and EGFR fluorescence intensities were calculated from these graphs and yielded values of 0.34 and 0.78 for cells transfected with the control siRNA and Netrin-1 siRNA, respectively, and 0.43 and 0.85 for cells transfected with the Netrin-1 plasmid and control plasmid, respectively (Fig 13D). These results suggest that Netrin-1 increases the amount and activation of EGFR at the level of the plasma membrane by impeding the internalization of this receptor. Netrin-1, therefore, fosters persistence of activated EGFR at the cell surface. This phenotypic alteration increases susceptibility of target cells to viral entry and, thus, leads to an even higher level of HCV infectivity than that induced by Netrin-1 at the level of the virions produced by infected cells.

## Discussion

In this study, we establish a causative relationship between a pro-oncogenic viral infection, namely HCV, and Netrin-1, an extensively studied dependence receptor ligand, in the context of cancer development. These observations were made in vitro as well as in chronically infected patients suffering from fibrotic liver disease, cirrhosis, or even HCC.

We report that the major molecular mechanism underlying the increase in Netrin-1 translation upon HCV infection is regulated by the NS5A and LARP1 proteins and that this increase is particularly marked in the microsomal compartments close to viral budding sites. In turn, we show that the rise in Netrin-1 leads to an increase in the level of infectivity of HCV particles, through both its presence on virions and its implication in the formation/morphogenesis of viral particles. Of note, LARP1 was recently identified as having a pivotal role in general [30,31,46,47] and in hepatic [48] carcinogenesis. Its implication in HCC now needs to be addressed in light of our findings regarding its sensitivity to an oncogenic virus, HCV, and its role in regulating Netrin-1.

The pro-viral effect mentioned above was further enhanced by the indirect effect of Netrin-1 on the persistence of EGFR at the surface of target cells, thus increasing their susceptibility to HCV viral entry. Indeed, Claudin-1/CD81 interactions, which enable HCV entry into hepatocytes [49], are mediated by the activation of EGFR [3,43]. As an HCV virion candidate component and an EGFR activator, Netrin-1 is, thereby, prone to favor the contribution of EGFRs to virion transfer from CD81 to CLDN1 at the level of the membrane [3]. EGFR signaling is implicated in HCC [7] and possibly in the resistance to the anti-HCC drug sorafenib [8]. The fact that Netrin-1 fosters HCV entry through fostering EGFR activation is in agreement with previous reports on the involvement of Netrin-1 in cancers [11,41,50], in which dysregulated EGFR expression and signaling play a major role [51].

As for high-throughput approaches and Netrin-1 biology, transcriptomic studies focusing on the regulation of Netrin-1 have so far not been reported. Although previous research has resulted in the identification of cell death-related signatures in HCV+ cells, in vitro, in either human or chimpanzee samples [52–58], most transcriptomic screens performed in the last 15 y on HCV-related samples were conducted using non-tiling arrays or RNAseq procedures. It is, therefore, not entirely surprising that Netrin-1, a difficult-to-target GC-rich transcript, has so far not been identified.

Our data revealed the occurrence of a positive feedback loop, represented here by the reciprocal induction of Netrin-1 and HCV. Such a positive feedback is relatively scarce in biological systems because of the irreversible imbalance this type of dynamics can rapidly produce. For this reason, our findings raise questions regarding the consequences of an increase in Netrin-1 on HCV infection, beyond HCV persistence per se. Although Netrin-1 expression did not confer a pro-survival advantage to cells used in this study, elevated levels of intrahepatic Netrin-1 may enhance the survival of hepatocytes previously altered and/or rendered more resistant to apoptosis by genetic or tissular injuries, such as HCV trans-acting factors or HCV-related chronic liver regeneration. Netrin-1-related enhancement of cell survival in a cytotoxic context has been observed in non-hepatic cancers following chemotherapy [59]. It is likely to be most relevant at the cirrhosis level, a harsh environment for hepatocytic survival, which displays the highest level of Netrin-1 signals, and even more so upon HCV infection. Importantly, previous studies demonstrated that the expression and activation of EGFR and its main dimerization partner HER-3 (ErbB-3) are frequently dysregulated in HCC [60–62]. Other investigations also indicated that EGFR and the dimerization inducer HER-3 may provide compensatory signals for cancer cells to escape targeted therapies in the liver [63]. Hence, our observation that EGFR undergoes functional upregulation by Netrin-1 indicates that it may mediate the potentially deleterious effects of Netrin-1 in liver pathologies. A relevant study on the role of Netrin-1 in fostering liver regeneration upon ischemia/reperfusion in mice livers [64] has been most recently published. We believe this study emphasizes the relevance of our work, since it concatenates the consequences of elevated Netrin-1 levels at the hepatic level with the Netrin-1 inducer status of HCV. Indeed, HCV may induce Netrin-1 for its own replicative benefit in a direct manner, but also in a liver maintenance prospective.

EGFR-mediated interferon resistance has been evidenced in the context of hepatitis C [65]. From the therapy perspective, it is widely accepted that treatments targeting HCV have undergone major improvements in recent years [66], although cirrhotic patients may in some cases remain more exposed to treatment failure than a majority of patients [67,68]. In this context, elevated levels of Netrin-1 in HCV+ cirrhotic patients should orient future research efforts not only in the direction of the onset of HCC but also of Netrin’s potential ability to condition the efficacy of direct-acting antivirals (DAAs) through a dysregulated Netrin-EGFR-endogenous interferon sensitive axis.

In summary, the HCV-Netrin-1 amplification loop studied herein is composed of two molecularly distinct Netrin-1-mediated arms, which converge toward a single phenotype of increased infectivity conferred to HCV particles. The involvement of Netrin-1 in hepatic pathobiology and the role of DR ligands in the persistence of infectious agents associated with cancer deserve further investigation.

## Materials and Methods

### Clinical Samples

Samples and PHHs were used according to the French IRB “Comité de Protection des Personnes (CPP) Sud-Est IV” agreement #11/040, obtained in 2011. Written informed consent was obtained from patients.

### Cell Culture and HCV Infection

PHHs were prepared, grown in William’s E medium, and infected with cell culture-adapted HCV as described previously [24]. The human hepatocyte cell line Huh7.5 was grown in DMEM (Life technologies) and supplemented with 10% fetal bovine serum (FBS; Thermo Scientific) and 1% penicillin-streptomycin (Life technologies). Twenty thousand cells per square centimeter were infected with HCV JFH1 [27] at an MOI of 0.1 (proliferative cells) or 0.05 (differentiated cells). In order to induce differentiation, 2% DMSO (Sigma) was added to the medium. Netrin-1-Fc (125 ng/mL) was obtained from Apotech Corp./Axxora. For *Netrin-1* mRNA stability assays, cells were treated with either DRB (25 μg/mL, Sigma-Aldrich) or actinomycin D (5 μg/mL, Sigma-Aldrich) in order to inhibit transcription for the various durations of time prior to total RNA isolation using the Extract-all reagent (Eurobio).

### Quantitative RT-PCR

Total RNA was extracted using the Nucleospin RNA/protein kit (Macherey-Nagel) for biopsies and the Extract-all reagent (Eurobio) for cultured cells. One μg of RNA was DNAse I-digested (Promega) and reverse transcribed with 5% DMSO, using the MMLV enzyme, according to the manufacturer’s instructions (Invitrogen). Real-time quantitative RT-PCR was performed on a LightCycler 480 device (Roche) using the iQ™ SYBR^®^Green Supermix (BIO-RAD). DMSO (10%, Sigma-Aldrich) was added to the PCR reaction for *Netrin-1* quantification. All PCR primer sequences and qPCR conditions are reported in the S2 Table.

### Isolation of Free Versus Membrane-Bound Polysomal RNAs and Nucleocytoplasmic Fractionation

The partitioning of *Netrin-1*, *GUS*, and *PMM1* mRNA between the cytosol (free polysomes) and ER membrane-bound polysome compartments was performed as described by Stephens et al. [69]. *GUS* and *PMM1* mRNAs were used to assess the quality of each fraction. *GUS* mRNA is membrane-enriched [70], whereas *PMM1* mRNA is not. Nucleocytoplasmic fractionation was performed as described previously [71].

### Plasmid Transfection

Two hundred thousand cells in ten-square-centimeter wells were transfected either with 2.5 μg Netrin-1 or with the neuronal vanilloid receptor VR1 expressing plasmid, or with the F-Luc-bearing Netrin-1 promoter reporter construct described previously [20] using the TransIT-LT1 reagent (Mirus Bio), following the manufacturer’s instructions.

ORF encoding LARP1 was picked from the Human ORFeome library (Open biosystems) and recombined into pGLuc vector (for luciferase tagging) [33] by gateway technology (Invitrogen). ORFs encoding HCV proteins were amplified from described vectors [72] and recombined into pGLuc vector and/or pFlag vector. Sequences of relevant primers are available upon request.

### Protein Complementation Assay

As described [33,34], combinations of plasmids encoding prey (A) and bait (B) proteins, each fused to a fragment of the *Gaussia princeps* luciferase protein (GLuc1 and GLuc2) or control vectors, were cotransfected into Huh-7.5 cells plated in 96-well plates in triplicates. At 24 hr post-transfection, cells were lysed and subjected to luciferase assays (Promega). Results were expressed as normalized luminescence ratios (NLR): the average luminescence signal in cells transfected with GLuc1-A and GLuc2-B divided by the average signal in wells transfected with GLuc1-A and an empty GLuc2 vector and those transfected with GLuc2-B and an empty GLuc1 vector. We benchmarked the sensitivity and accuracy of this screen by including a random reference set (RRS) composed of 53 noninteracting human protein pairs and a set of host factors known to interact with various HCV proteins [33].

### Density Gradient Analysis

Cell culture supernatants collected at the indicated time points were loaded on top of 10%–60% sucrose gradients and centrifuged at 38,000 rpm at 4°C for 16 h in a SW41 rotor (Beckman). A total of 12 fractions (1 ml each) were collected and their densities were determined with a refractometer (Euromex). HCV RNA in each fraction (150 μl) was extracted with the Nucleospin RNA Virus kit (Macherey-Nagel) and quantified by RT-qPCR. The infectivity of HCV virions in each fraction was determined on Huh7.5 cells using the TCID_50_ protocol [73] as described below.

### siRNA-Mediated Knockdown

Twenty thousand cells per square centimeter were transfected with various concentrations (12.5, 25, and 50 nM final concentration) of a nontargeting control siRNA, Netrin-1 siRNA, LARP1 siRNA, or EGFR siRNA (Sigma-Aldrich) using Lipofectamine 2000 (Invitrogen), according to the manufacturer’s instructions. siRNAs sequences are listed in S3 Table.

### HCV Infectivity Assay

The level of infectivity of the HCV produced in cell culture was measured following the TCID_50_ protocol outlined by Lindenbach [73]. A human HCV antiserum at a 1/500 dilution (initially validated against the anti-HCV capsid C7/50 clone (Abcam) in a double immunofluorescence assay) and a goat anti-human Alexa 488 secondary antibody (Invitrogen) were used, at a concentration of 1 μg/mL. Cells were counterstained with DAPI and examined with a Nikon TE-2000E epifluorescence microscope. Titers were calculated using the Reed and Muench method [73].

### RNA and Protein Immunoprecipitation

RIP-Chip was performed as described in Keene et al. [74], using control IgG and anti-LARP1 antibodies (Novus Biologicals) and protein G magnetic beads (Millipore) prior to RNA extraction and RT-qPCR. For direct or indirect protein immunoprecipitation, Huh7.5 cells were lysed in RIPA extraction buffer. Lysates were incubated with anti-LARP1 antibody at 4°C for 1 h. Protein G magnetic beads were then added to the antibody/lysate mixture and incubated for 45 min before immunoblotting.

### Cell Death and Proliferation Assays

Caspase-3 activity assays were performed using the Caspase 3/CPP32 Colorimetric Assay Kit, according to manufacturer’s instructions (Gentaur Biovision). The cell proliferation assay was performed using neutral red uptake as described by Repetto et al. [75].

### Immunohistochemistry

Formalin-fixed, paraffin-embedded liver samples were sectioned at a thickness of 4 μm. After deparaffinisation and rehydration, tissue sections were unmasked in citrate buffer (pH 9) in a 96°C water bath for 50 min. To block endogenous peroxidases, slides were incubated in 5% hydrogen peroxide in sterile water. Slides were then incubated at room temperature for 1 h with a polyclonal goat antibody recognizing human Netrin-1 (R&D Systems), diluted 1/800 in an antibody diluent solution (Dako). For HCV immunostaining, slides were incubated overnight at 4°C with a human anti-HCV E2 antibody [23] diluted 1/50. After three washes in PBS, slides were incubated with a biotinylated secondary antibody bound to a streptavidin peroxidase conjugate (Lsab+ kit, Dako). Netrin-1 and HCV staining were contextualized by DAB staining. Nuclei were counterstained with hemalun.

### HCVpp Infection and Luciferase Assay

HCVpp and their VSV-G and Env-negative controls were produced as described previously [44]. A construct package containing the Renilla luciferase gene under the control of the cytomegalovirus (CMV) promoter (pMLVΨCMV-Luc) was used as a reporter. Huh7.5 cells transduced with virions containing the luciferase transgene were lysed 3 d post-infection, and luciferase activity was monitored by using the *Renilla* luciferase assay system according to the manufacturer’s instructions (Promega).

### FACS Analyses

Huh7.5 cells were detached with Versene buffer, washed in PBS and centrifuged at 1 200 r.p.m for 5 min. Cells were then stained with an EGFR antibody (Calbiochem) at a 1/1000 dilution and a mouse anti-human Alexa 488-conjugated secondary antibody (Invitrogen). EGFR membrane expression was analyzed using a FACscalibur (BD).

### Immunoblotting

Immunoblotting was performed using standard protocols with antibodies against the HA-tag (Sigma-Aldrich), actin (Sigma-Aldrich), total EGFR (Millipore), phosphorylated (position 1068) EGFR (Cell Signaling), Netrin-1 (R&D System), core (Abcam), HSP60 (Bd Biosciences), and LARP1 (Novus).

### Immunofluorescence

Cells were fixed in 4% paraformaldehyde for 10 min and permeabilized for 30 min in 0.15% Triton-X100 in PBS + 3% BSA. Incubation with primary antibodies was performed for 2 hrs in 0.15% Triton-X100 in PBS + 3% BSA at room temperature. dsRNA, EGFR, and EEA1 immunolocalization was performed using anti-dsRNA J2 (Scicons) [76], anti-EEA1, anti-Calnexin, anti-Puromycin (BD Biosciences), anti-LARP1 (Novus Biologicals), anti-NS5A 9E10 (gift from C.Rice), and anti-EGFR (Millipore) antibodies (2 μg/mL). For puromycylation assays, 200 μM emetin (Sigma) and 90 μM puromycin (Sigma) were incubated on cells for 10 min before harvest. Samples were incubated with Alexa-488-goat anti-mouse and Alexa-594-goat anti-rabbit antibodies (Invitrogen, 1 μg/mL) for 1 h at room temperature. Nuclei were counterstained with Hoechst 33342. Images were acquired using a Leica SP5X confocal microscope equipped with LAS AF software. Subsequent analyses were performed using the JACop ImageJ colocalization plugin (http://rsb.info.nih.gov/ij/plugins/track/jacop.html) and its plot profile function.

### Virus Particle Neutralization Assay

Naïve Huh7.5 cells were transfected with Netrin-1 or VR1-expressing plasmids and infected with HCV for 4 d. The supernatant was first clarified at 8,000 g for 15 min, then ultracentrifugated on a 20% sucrose / 1X TNE cushion for 4 h, minimizing the carryover of soluble, non-viral material. The pelleted virions were collected in fresh culture medium and incubated with two distinct anti-Netrin-1 antibodies (clones #2F5 from Netris Pharma and #AF1009 from R&D Systems) or DCC-Fc (recombinant receptor of Netrin-1 from R&D Systems) overnight, at a concentration of 10 μg/ml. To quantify the level of viral infectivity, we performed a TCID_50_ assay following the TCID_50_ protocol. Naïve Huh7.5 cells were seeded in a 96-well plate at a density of 20,000 cells per square centimeter and infected the day after with the neutralized virions at serial dilutions of 10^−1^ to 10^−8^ (one dilution per line). Hence, each biological sample was processed in the context of 12 technical replicates. Immunofluorescence staining was performed 3 d post-infection using a human HCV antiserum at a 1/500 dilution and a goat anti-human Alexa 488 secondary antibody at a concentration of 1 μg/mL. Positive wells were counted with a Nikon TE-2000E epifluorescence microscope and titers were calculated using the adapted Reed and Muench method [77] as considered by Lindenbach [73].

## Supporting Information

S1 Data Compiled individual quantitative observations. (XLSX)

S1 Fig Correlation between Netrin-1 and HCV sero/genotype (**A**), age (**B**), etiology (**C**). **D**. *Netrin-1* mRNA expression in alcohol-induced fibrosis and HCV-induced fibrosis. The underlying data for panels in this figure can be found in S1 Data.
(EPS)

S2 Fig Immunoprecipitation of LARP1. Immunoblotting using anti-LARP1 antibody after immunoprecipitation of LARP1 (control of purification).
(EPS)

S3 Fig Assessment of *LARP1* mRNA knockdown. Cells were processed for LARP1 protein quantification by immunoblotting. The underlying data for panels in this figure can be found in S1 Data.
(TIF)

S4 Fig HCV RNA release upon Netrin-1 overexpression in vitro. Huh7.5 cells were transfected with a plasmid encoding Netrin-1 or the neuronal vanilloid receptor VR1 as a control and infected at a MOI of 0.1 the day after seeding. HCV RNA was quantified by RT-qPCR in each collected fraction after sucrose density gradient fractionation, 3 d post-infection (*n* = 2). Buoyant densities were determined by refractometry (*n* = 3). The underlying data for panels in this figure can be found in S1 Data.
(EPS)

S5 Fig Netrin-1 does not exert its pro-HCV effect through protection against cell death. Caspase-3 activity (**A**) and neutral red (**B**) assays were performed after Netrin-1 plasmid transfection in Huh7.5 cells (*n* = 2). The underlying data for panels in this figure can be found in S1 Data.
(EPS)

S6 Fig Netrin-1 increases HCV RNA and propagation in vitro. Recombinant soluble Netrin-1-Fc was added to the medium of Huh7.5 cells 12 h before infection. Intracellular HCV RNA was quantified by RT-qPCR at each time point (**A**–**B**), while supernatant infectivity was quantified on indicated days post-infection by the TCID_50_ method (**C**,**D**) in proliferative (**A,C**) or differentiated (**B,D**) cells (data shown as mean ± standard deviation, *n* = 3, Wilcoxon test, *p* < 0.05). The underlying data for panels in this figure can be found in S1 Data.
(EPS)

S7 Fig Caspase-3 activity and cell viability are not altered by Netrin-1-Fc treatment. Cultures of proliferative and differentiated Hu7.5 cells were exposed to Netrin-1-Fc 12 h before infection and then harvested at the indicated time points. Protein extracts from proliferative (**A**) and differentiated (**B**) cells were submitted to intracellular cleaved caspase-3 ELISA assays. Caspase-3 activity levels in Netrin-1-Fc treated samples and control samples are shown (as mean ± standard deviation, *n* = 3). **C**. Cell proliferation levels are not altered by Netrin-1-Fc treatment in proliferative Huh7.5 cells, irrespective of their infection status. **D**. Viability of proliferative Huh7.5 cells is not altered by treatment with increasing doses of Netrin-1-Fc, irrespective of their infection status. The underlying data for panels in this figure can be found in S1 Data.
(EPS)

S8 Fig Netrin-1-FLAG increases HCV in vitro. Recombinant soluble Netrin-1-FLAG was added to the medium of Huh7.5 cells 12 h before infection. Intracellular HCV RNA was quantified by RT-qPCR at each time point (Wilcoxon test, *p* < 0.05). The underlying data for panels in this figure can be found in S1 Data.
(EPS)

S9 Fig HCV RNA secretion upon Netrin-1 RNAi-based knockdown in vitro. Huh7.5 cells were transfected with a Netrin-1 siRNA or a nontargeting (control) siRNA, infected at a MOI of 0.1 24 h after seeding and trypsinized 5 d post-infection before a second siRNA transfection. Culture supernatants were collected 6 d post-infection and subjected to sucrose gradient centrifugation. HCV RNA in gradient fractions was quantified by RT-qPCR. Buoyant densities were determined by refractometry (*n* = 3). The underlying data for panels in this figure can be found in S1 Data.
(EPS)

S10 Fig Netrin-1 does not exert its pro-HCV effect through protection against cell death. Caspase-3 activity (**A**) and neutral red (**B**) assays were performed after Netrin-1 siRNA transfection (*n* = 2). The underlying data for panels in this figure can be found in S1 Data.
(EPS)

S11 Fig Anti-Netrin-1 blocking antibody impedes HCV RNA and propagation in vitro. The 2F5 anti-Netrin-1 monoclonal antibody or the H4 irrelevant monoclonal antibody was added to the medium at the time of seeding and renewed every 2 d. Huh7.5 cells were infected at an MOI of 0.1. Intracellular HCV RNA was quantified by RT-qPCR at each time point (**A**). Supernatant infectivity was quantified on indicated days post-infection by the TCID_50_ method (**B**). Data are represented as mean ± standard deviation (*n* = 3, Wilcoxon test, *p* < 0.05). The underlying data for panels in this figure can be found in S1 Data.
(EPS)

S12 Fig Netrin-1 does not affect HCV replication per se. Replicon-bearing cells [40] were treated with Netrin-1-Fc every 3 d and harvested daily for intracellular RNA extraction and HCV RNA quantification by RT-qPCR. **A.** Proliferative cells were trypsinized every 3 d. **B.** Differentiated cells were treated with DMSO 3 d after seeding prior to Netrin-Fc treatment. The underlying data for panels in this figure can be found in S1 Data.
(EPS)

S13 Fig Expression of Netrin-1 receptor transcripts in Huh7.5 cells. The mRNAs coding for receptors UNC5A/B/C/D and DCC were quantified by RT-qPCR in naïve Huh 7.5 cells, PHH (*n* = 4) and uninfected liver tissues (*n* = 6) after 5 d of culture (data are shown as mean ± standard deviation, *n* = 2). The underlying data for panels in this figure can be found in S1 Data.
(EPS)

S14 Fig UNC5A modulates HCV in vitro. Huh7.5 cells were infected with HCV genotype 2 strain during 4 d, and *UNC5A* mRNA was quantified by RT-qPCR in proliferative cells (**A**) or differentiated cells (**B**) (mean ± standard deviation, *n* = 3 indep. expts, two-tailed Mann-Whitney test, *p* < 0.05). Huh7.5 cells were transfected with the VR1-HA or UNC5A-HA plasmids and infected with HCV genotype 2 strain over 4 d. **C**. Intracellular HCV RNA was quantified by RT-qPCR. **D**. Intracellular infectivity was quantified likewise (*n* = 3 indep. expts, two-tailed Mann-Whitney test, *p* < 0.05). Huh7.5 cells were transfected with UNC5A siRNA and infected as before. **E**. Intracellular HCV RNA was quantified by RT-qPCR. **F**. Supernatant infectivity was quantified (TCID_50_ method, *n* = 3 indep. expts, two-tailed Mann-Whitney test, *p* < 0.05). The underlying data for panels in this figure can be found in S1 Data.
(EPS)

S15 Fig Netrin-1 modulates EGFR activation at the level of the plasma membrane in EGF-synchronized cells. Serum-starved (24 h) Huh7.5 cells were transfected with Netrin-1 siRNAs or a Netrin-1 expressing plasmid and infected at a MOI of 0.1 the day after seeding in presence of serum for 24 h. Cells were collected 3 d post-infection and analyzed by FACS using an EGFR antibody (**A**) specific for an extracellular epitope of the protein (data represented as mean ± standard deviation, *n* = 5). **B**. Netrin-1 modulates EGFR phosphorylation. Huh7.5 cells were transfected and infected as in (**A**). **C**. Netrin-1 modulates EGFR phosphorylation. Cells were lysed 3 d post-infection and analyzed by immunoblotting using anti-EGFR, anti-phospho-EGFR, and anti-β-actin antibodies (*n* = 3). The underlying data for panels in this figure can be found in S1 Data.
(EPS)

S16 Fig Netrin-1 modulates EGFR activation at the plasma membrane. Huh7.5 cells were transfected with Netrin-1 siRNAs or a Netrin-1-expressing plasmid and infected at a MOI of 0.1 the day after seeding. Cells were collected 3 d post-infection and analyzed by FACS using an EGFR antibody (**A**) or a CD81 antibody (**B**), both specific for an extracellular epitope of the protein (data represented as mean ± standard deviation, *n* = 5). **C**. Netrin-1 modulates EGFR phosphorylation. Huh7.5 cells were transfected and infected as in (A). Cells were lysed 3 d post-infection and analyzed by immunoblotting using anti-EGFR, anti-phospho-EGFR, and anti-β-actin antibodies (*n* = 3). The underlying data for panels in this figure can be found in S1 Data.
(EPS)

S17 Fig Internalization of EGFR upon acute EGF stimulation. HCV-infected Huh7.5 cells were subjected to Netrin-1 knockdown or forced expression as described previously. **A**. EGFR mRNA quantification by RT-qPCR. **B**. Total EGFR quantification by immunoblotting. **C**. Plasma membrane-located EGFR quantification by flow cytometry. Cells were analyzed using an EGFR antibody that recognizes an extracellular epitope of the protein. EGF was added for 0, 5, or 15 min prior to sample processing. The underlying data for panels in this figure can be found in S1 Data.
(TIF)

S1 Supporting Information HCV immunostaining. Taking into account HCV staining methods depicted in Galy et al. 2009 [78] and Ballardini et al.1995 [79], and in order to provide a more robust set of results regarding the technical validation of HCV staining, we performed immunostains on liver tissues with a previously neutralized anti-E2 antibody using an Huh7.5-infected cell lysate. The neutralization of the antibody was tested first on Huh7.5 cells by immunofluorescence. A. Immunofluorescence staining on Huh7.5 cells using an isotype control (clone RO4), an anti-E2 (clone CBH5 [23]), and the same neutralized anti-E2 Ig. The E2 antibody (1 μg) was pre-incubated for 24 h with Huh7.5 cell lysates infected or not infected with the HCV JFH1 strain for 5 d. Immunostaining on HCV-/+ Huh7.5 cells was then performed. As shown on the right picture, no E2-staining was observed with the neutralized antibody, allowing one to implement the same strategy on tissue sections. B. Immunostaining on liver tissues (same samples as previously used in the first version of the study) using an isotypic control (clone RO4) and the same anti-E2 (clone CBH5) previously neutralized or not as depicted above. E2-staining was essentially abolished in tissue sections in which the neutralized antibody was used. Our results therefore confirm the specificity of the HCV protein staining. C. In order to provide more insight on the cell types stained by HCV and Netrin-1 Abs, finer observation of the processed tissues is shown after interpretation by a pathologist (M.D.S.). Netrin-1 expression was analyzed in a cirrhotic patient by immunohistochemistry with an antibody against Netrin-1. Netrin-1 immunostaining was detected in the cytoplasm of hepatocytes (H), while inflammatory cells and biliary epithelium (BE) are negative. The HCV protein expression was analyzed in the same tissue sample using an antibody against E2. HCV is expressed in the cytoplasm of hepatocytes, while no staining can be found in the biliary epithelium. Globally homogenous intensity of Netrin-1 staining was observed in hepatocytes, while HCV staining underwent important variations from one area to the other. This suggests that bystander effects conveyed through paracrine or soluble mediators are in part implicated in Netrin-1 induction upon HCV infection in patients.
(TIF)

S2 Supporting Information HCV and cirrhosis cooperate to induce Netrin-1. As HCV infection and cirrhosis are prominent risk factors for HCC, we investigated whether HCV-induced Netrin-1 was particularly elevated in cirrhotic patients. In addition, *Netrin-1* mRNA levels were significantly further elevated in HCV-infected cirrhotic patients compared to their HCV-negative cirrhotic counterparts (F4: 4-fold, *p* < 0.0001; S2A Supporting Information). The increase in Netrin-1 mRNA levels was even more evident in HCV(+) cirrhotic patients compared to HCV-negative, non-cirrhotic (F0–F3) samples (>30-fold, *p* < 0.0001, S2A Supporting Information). We then quantified *Netrin-1* mRNA levels in liver biopsies from a cohort of HCV(-) and HCV(+) patients with HCC. Tumor samples exhibited a decrease in *Netrin-1* mRNA compared with all cirrhosis samples, which could be due to the overall decrease in HCV levels (S2B Supporting Information) [80]. However, *Netrin-1* mRNA was found to be moderately but still significantly increased (1.4-fold, *p* = 0.03) in HCV-related HCC compared to HCV-unrelated HCC, a result again supporting a connection between HCV infection and Netrin-1 expression throughout HCV pathophysiology (S2B Supporting Information). Importantly, a comparison of HCV(-) biopsies revealed that HCV negative cirrhosis (i.e., F4) samples already displayed a 4-fold to 12-fold increase in *Netrin-1* mRNA compared to all other HCV-negative samples (respectively, F0: 10-fold, *p* < 0.0001; F1: 4-fold, *p* = 0.02; F2: 9-fold, *p* < 0.0001; F3: 12-fold, *p* < 0.0001, Fig 12D). In addition, HCV(-) HCC had higher levels of Netrin-1 compared with all fibrosis categories (respectively, F0: 8-fold, *p* < 0.0001; F2: 7-fold, *p* < 0.0001; F3: 10-fold, *p* < 0.0001, S2C Supporting Information). Taken together, these data indicate that HCV and cirrhosis cooperate in inducing Netrin-1.
(TIF)

S3 Supporting Information Dose response assay of siRNA-based Netrin-1 depletion on HCV. Huh7.5 cells were transfected with a Netrin-1 siRNA or a nontargeting (control) siRNA at different concentrations and infected with HCV at a MOI of 0.1 24 h after seeding. Intracellular HCV RNA was quantified by RT-qPCR 3 d post-infection. As shown here, there was a correlation between Netrin-1 siRNA-mediated effects and its knockdown efficiency, indicating that the silencing effect was dose-dependent.
(TIF)

S1 Table Characteristics of patient cohort. Clinical, biological, and virological parameters of virus-free, HBV+, and HCV+ patients are indicated. The study protocol was approved by the French IRB “Comité de Protection des Personnes (CPP) Sud-Est IV” (#11/040). Written informed consent was received from patients.
(DOCX)

S2 Table Oligonucleotides and PCR conditions. (DOCX)

S3 Table siRNA sequences. (DOCX)

S1 Text Microscopy. (DOCX)

## Abbreviations

DAA: direct-acting antiviral
DCC: deleted in colorectal cancer
DMSO: dimethyl sulfoxide
DR: dependence receptor
EGFR: epidermal growth factor receptor
ER: endoplasmic reticulum
GUS: glucuronidase
HA: hemagglutinin
HBV: hepatitis B virus
HCC: hepatocellular carcinoma
HCV: hepatitis C virus
HCVpp: heptatitis C virus pseudoparticles
ICCB: intensity correlation coefficient-based
JFH1: japanese fulminant hepatitis 1
LARP1: La-related protein 1
NLR: normalized luminescence ratio
ORF: open reading frame
PCA: protein-fragment complementation assay
PHH: primary human hepatocytes
PMM1: phosphomannomutase 1
RPS18: ribosomal protein S18
TOP: terminal oligopyrimidine tract
UNC5: uncoordinated receptor-5
VR1-HA: HA-tagged vanilloid receptor
