# Supplementary figures and images for: Epidermal Growth Factor Receptor-Dependent Mutual Amplification between Netrin-1 and the Hepatitis C Virus
Source: PLoS Biol. 2016 Mar 31;14(3):e1002421. doi: 10.1371/journal.pbio.1002421 (PMC4816328; doi:10.1371/journal.pbio.1002421)

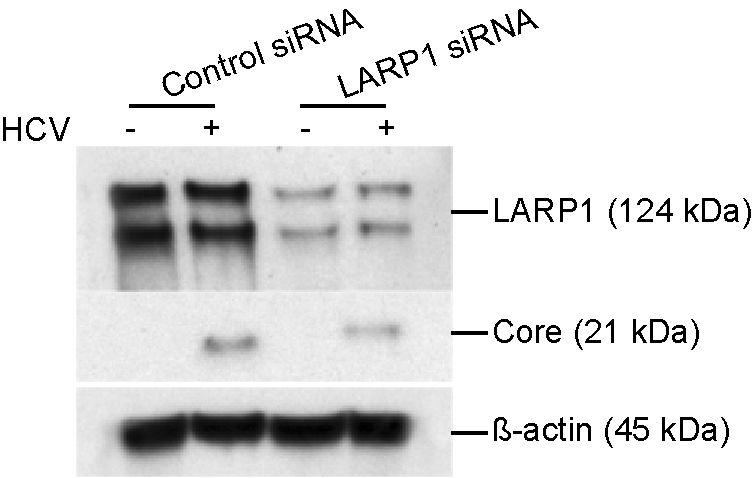

Supplement: S3 Fig — Cells were processed for LARP1 protein quantification by immunoblotting. The underlying data for panels in this figure can be found in S1 Data. (TIF) [file pbio.1002421.s004.tif]

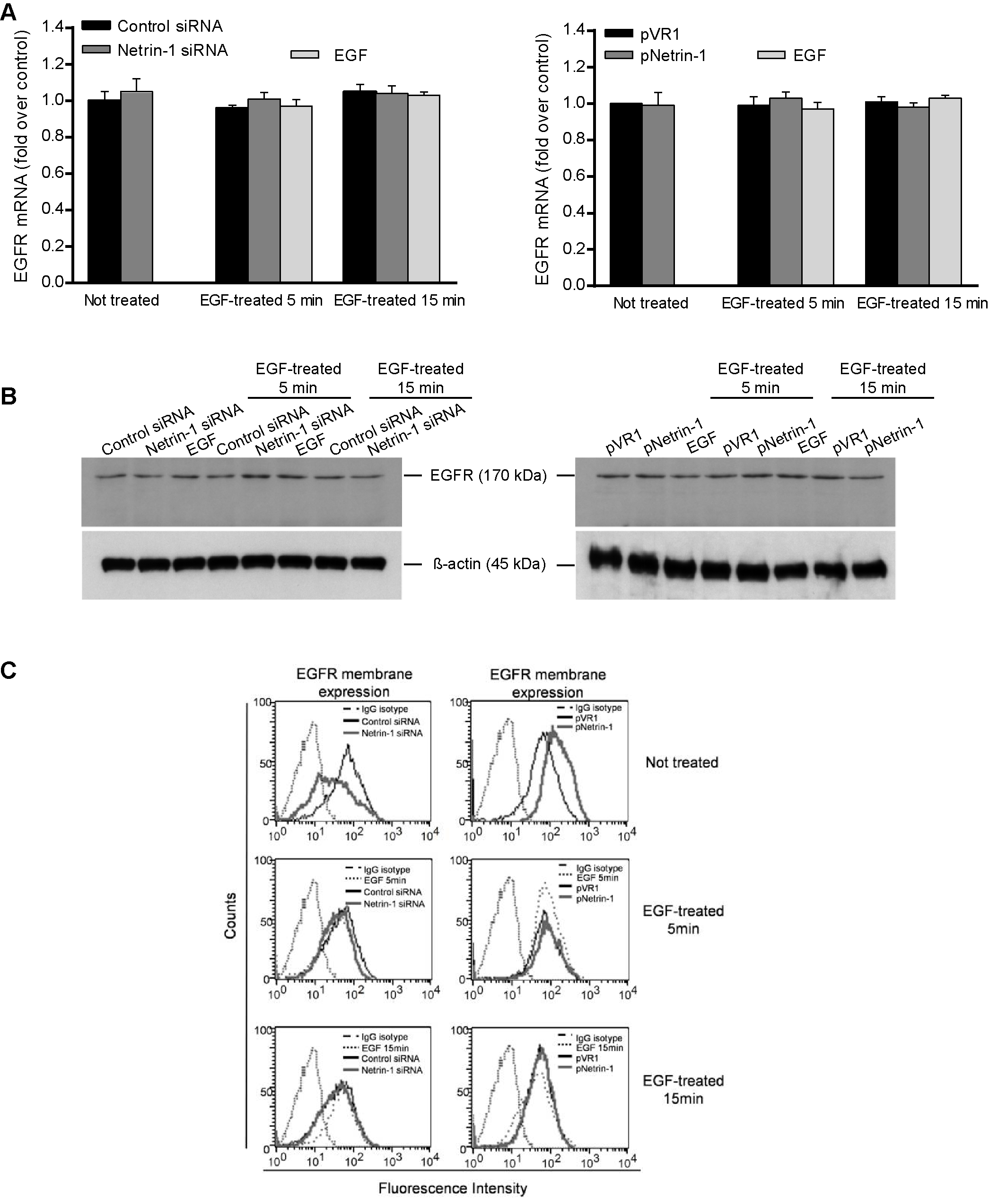

Supplement: S17 Fig — HCV-infected Huh7.5 cells were subjected to Netrin-1 knockdown or forced expression as described previously. A. EGFR mRNA quantification by RT-qPCR. B. Total EGFR quantification by immunoblotting. C. Plasma membrane-located EGFR quantification by flow cytometry. Cells were analyzed using an EGFR antibody that recognizes an extracellular epitope of the protein. EGF was added for 0, 5, or 15 min prior to sample processing. The underlying data for panels in this figure can be found in S1 Data. (TIF) [file pbio.1002421.s018.tif]

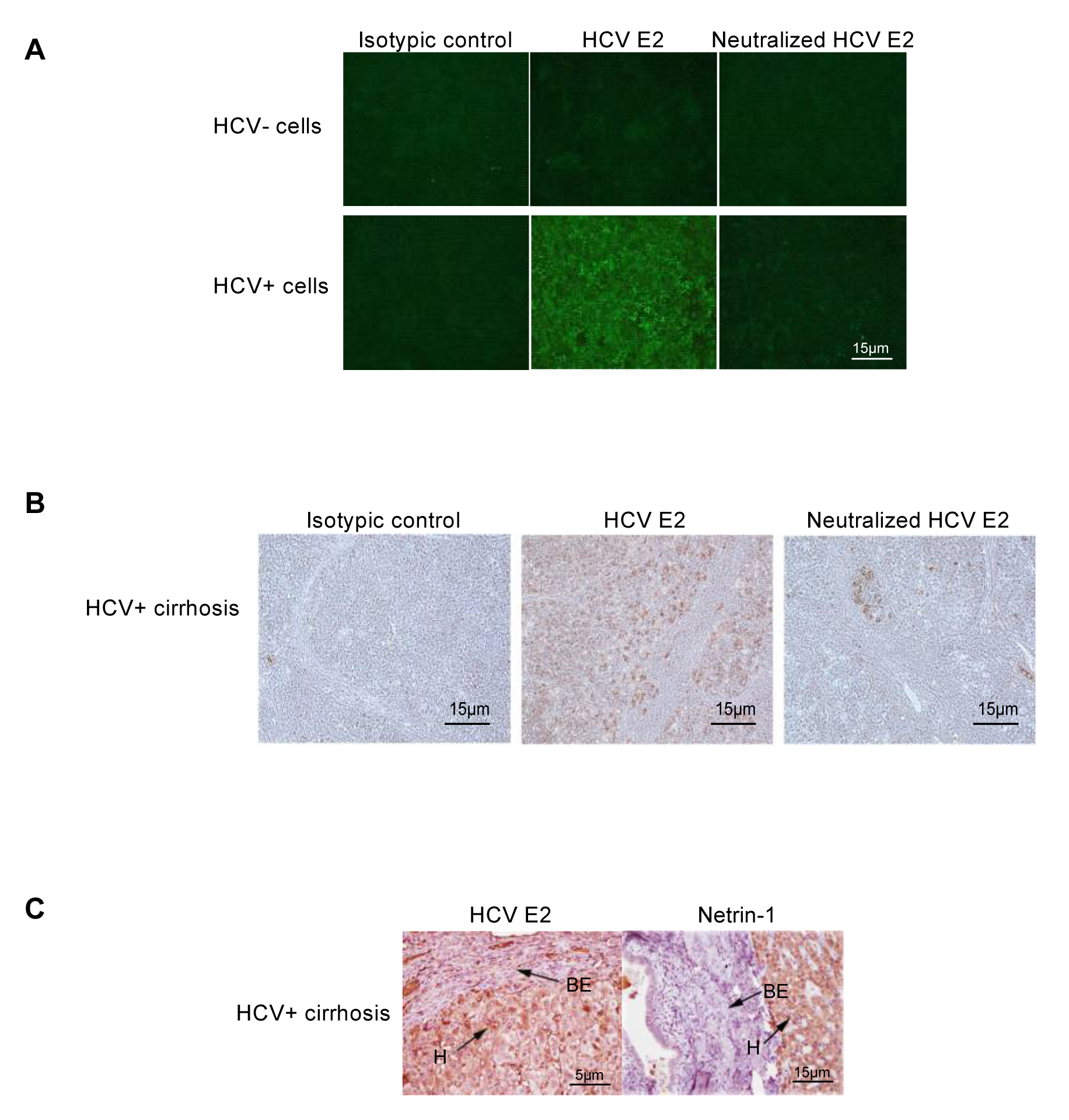

Supplement: S1 Supporting Information — Taking into account HCV staining methods depicted in Galy et al. 2009 [78] and Ballardini et al.1995 [79], and in order to provide a more robust set of results regarding the technical validation of HCV staining, we performed immunostains on liver tissues with a previously neutralized anti-E2 antibody using an Huh7.5-infected cell lysate. The neutralization of the antibody was tested first on Huh7.5 cells by immunofluorescence. A. Immunofluorescence staining on Huh7.5 cells using an isotype control (clone RO4), an anti-E2 (clone CBH5 [23]), and the same neutralized anti-E2 Ig. The E2 antibody (1 μg) was pre-incubated for 24 h with Huh7.5 cell lysates infected or not infected with the HCV JFH1 strain for 5 d. Immunostaining on HCV-/+ Huh7.5 cells was then performed. As shown on the right picture, no E2-staining was observed with the neutralized antibody, allowing one to implement the same strategy on tissue sections. B. Immunostaining on liver tissues (same samples as previously used in the first version of the study) using an isotypic control (clone RO4) and the same anti-E2 (clone CBH5) previously neutralized or not as depicted above. E2-staining was essentially abolished in tissue sections in which the neutralized antibody was used. Our results therefore confirm the specificity of the HCV protein staining. C. In order to provide more insight on the cell types stained by HCV and Netrin-1 Abs, finer observation of the processed tissues is shown after interpretation by a pathologist (M.D.S.). Netrin-1 expression was analyzed in a cirrhotic patient by immunohistochemistry with an antibody against Netrin-1. Netrin-1 immunostaining was detected in the cytoplasm of hepatocytes (H), while inflammatory cells and biliary epithelium (BE) are negative. The HCV protein expression was analyzed in the same tissue sample using an antibody against E2. HCV is expressed in the cytoplasm of hepatocytes, while no staining can be found in the biliary epithelium. Globally homogenous inte [file pbio.1002421.s019.tif]

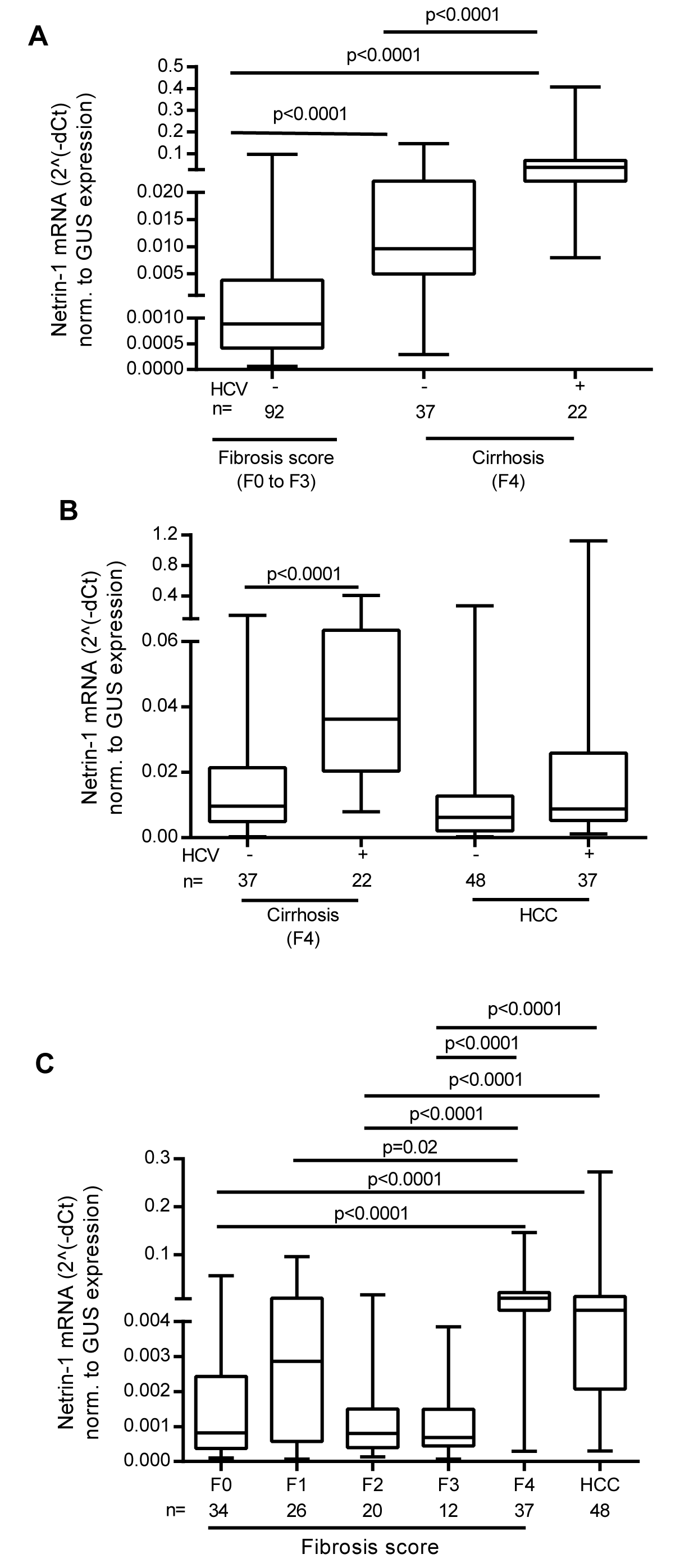

Supplement: S2 Supporting Information — As HCV infection and cirrhosis are prominent risk factors for HCC, we investigated whether HCV-induced Netrin-1 was particularly elevated in cirrhotic patients. In addition, Netrin-1 mRNA levels were significantly further elevated in HCV-infected cirrhotic patients compared to their HCV-negative cirrhotic counterparts (F4: 4-fold, p < 0.0001; S2A Supporting Information). The increase in Netrin-1 mRNA levels was even more evident in HCV(+) cirrhotic patients compared to HCV-negative, non-cirrhotic (F0–F3) samples (>30-fold, p < 0.0001, S2A Supporting Information). We then quantified Netrin-1 mRNA levels in liver biopsies from a cohort of HCV(-) and HCV(+) patients with HCC. Tumor samples exhibited a decrease in Netrin-1 mRNA compared with all cirrhosis samples, which could be due to the overall decrease in HCV levels (S2B Supporting Information) [80]. However, Netrin-1 mRNA was found to be moderately but still significantly increased (1.4-fold, p = 0.03) in HCV-related HCC compared to HCV-unrelated HCC, a result again supporting a connection between HCV infection and Netrin-1 expression throughout HCV pathophysiology (S2B Supporting Information). Importantly, a comparison of HCV(-) biopsies revealed that HCV negative cirrhosis (i.e., F4) samples already displayed a 4-fold to 12-fold increase in Netrin-1 mRNA compared to all other HCV-negative samples (respectively, F0: 10-fold, p < 0.0001; F1: 4-fold, p = 0.02; F2: 9-fold, p < 0.0001; F3: 12-fold, p < 0.0001, Fig 12D). In addition, HCV(-) HCC had higher levels of Netrin-1 compared with all fibrosis categories (respectively, F0: 8-fold, p < 0.0001; F2: 7-fold, p < 0.0001; F3: 10-fold, p < 0.0001, S2C Supporting Information). Taken together, these data indicate that HCV and cirrhosis cooperate in inducing Netrin-1. (TIF) [file pbio.1002421.s020.tif]

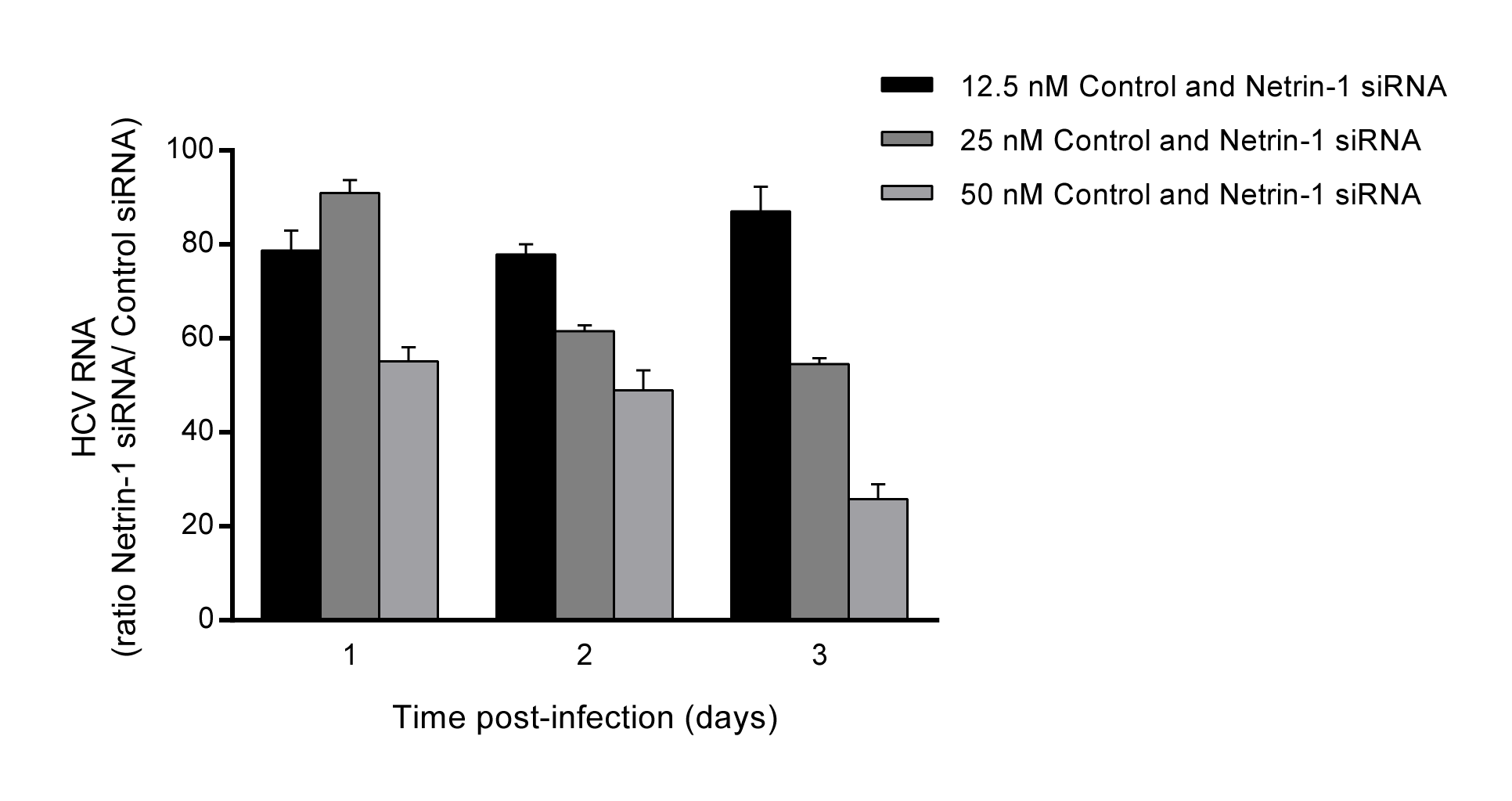

Supplement: S3 Supporting Information — Huh7.5 cells were transfected with a Netrin-1 siRNA or a nontargeting (control) siRNA at different concentrations and infected with HCV at a MOI of 0.1 24 h after seeding. Intracellular HCV RNA was quantified by RT-qPCR 3 d post-infection. As shown here, there was a correlation between Netrin-1 siRNA-mediated effects and its knockdown efficiency, indicating that the silencing effect was dose-dependent. (TIF) [file pbio.1002421.s021.tif]
