# Supplementary material for: Epidermal Growth Factor Receptor-Dependent Mutual Amplification between Netrin-1 and the Hepatitis C Virus
Source: PLoS Biol. 2016 Mar 31;14(3):e1002421. doi: 10.1371/journal.pbio.1002421 (PMC4816328; doi:10.1371/journal.pbio.1002421)
Supplement: S2 Table — (DOCX) [file pbio.1002421.s023.docx]

| **Target name** | **Genbank accession number** | **primers sequences** | **PCR conditions** | **amplicon length (bp)** |
| --- | --- | --- | --- | --- |
| *Netrin-1*  *(NTN1)* | NM_004822 | For CTTCTGCGGCAGGCGGACAGAT  Rev ACGCGTTGCAGAGGTGGCACGA | denaturation 95°C  annealing 60°C  extension 72°C  (qPCR mix supplemented with 10% DMSO) | 385 |
| *EGFR* | AF288738 | For CTCCAGGAAGCCTACGTGAT  Rev GTCTTTGTGTTCCCGGACAT | denaturation 95°C  annealing 59°C  extension 72°C | 300 |
| *HCV* | AB559564 | For GTCTAGCCATGGCGTTAGTA Rev CTCCCGGGGCACTCGCAAGC | denaturation 95°C  annealing 60°C  extension 72°C | 246 |
| *GUS* | NM_001293105 | For CGTGGTTGGAGAGCTCATTTGGAA  Rev TTCCCCAGCACTCTCGTCGGT | denaturation 95°C  annealing 60°C  extension 72°C | 72 |
| *LARP1* | [NM_015315.4](http://www.ncbi.nlm.nih.gov.gate2.inist.fr/nuccore/NM_015315.4) | For GCCTGGCAACCAGAGATCAAA  Rev TCAAACTTTCGGTAGCCAAACT | denaturation 95°C  annealing 55°C  extension 72°C | 194 |
| *RPS18* | [NM_022551.2](http://www.ncbi.nlm.nih.gov.gate2.inist.fr/nuccore/NM_022551.2) | For ATCACCATTATGCAGAATCCACG  Rev GACCTGGCTGTATTTTCCATCC | denaturation 95°C  annealing 55°C  extension 72°C | 93 |

**Supplementary Table 2**
