# Supplementary material for: Epidermal Growth Factor Receptor-Dependent Mutual Amplification between Netrin-1 and the Hepatitis C Virus
Source: PLoS Biol. 2016 Mar 31;14(3):e1002421. doi: 10.1371/journal.pbio.1002421 (PMC4816328; doi:10.1371/journal.pbio.1002421)
Supplement: S3 Table — (DOCX) [file pbio.1002421.s024.docx]

| **Target transcript** | **siRNA sequence 5’-3’** |
| --- | --- |
| *Netrin-1* | AAGCUGGACGCAGCAUGAUGC |
| *EGFR 3* | CAUCCAAUUUAUCAAGGAATT |
| *EGFR 4* | GGAACUGGAUAUUCUGAAATT |

**Supplementary Table 3**
