# Supplementary material for: Epidermal Growth Factor Receptor-Dependent Mutual Amplification between Netrin-1 and the Hepatitis C Virus
Source: PLoS Biol. 2016 Mar 31;14(3):e1002421. doi: 10.1371/journal.pbio.1002421 (PMC4816328; doi:10.1371/journal.pbio.1002421)
Supplement: S1 Text — (DOCX) [file pbio.1002421.s025.docx]

**Supporting Text 1**

**Microscopy.**

As stated in Bolte et al. [1], the work of Li et al. [2] is of interest in the search for an interpretable representation of colocalization. It is assumed that the overall difference of pixel intensities from the mean intensity of a single fluorophore is equal to zero, ∑ _n pixels_ (Ai-a) = 0 and ∑ _n pixels_ pixels (Bi-b) = 0 with the upper-case character being the current pixel's intensity and the lower-case character being the current channel's mean intensity. As a consequence, the product of the two equalities should tend to zero. Considering colocalizing pixels, this product should be positive as each difference from the mean is of the same sign. The differences of intensities between both channels are scaled down by fitting the histogram of both images to a 0-1 scale. The intensity correlation analysis results are then presented as a set of two parameters, each showing the normalized intensities (from 0 to 1) as a function of the product (Ai-a)(Bi-b) for each channel.

As a consequence, IF colocalization figures depict four items:

- A representative Li diagram of the intensity correlation analyses performed from each acquisition of a given biological sample in which (i) exclusion is depicted at the pixel level on the negative (left) zone of the diagram and (ii) colocalization is depicted at the pixel level on the postive (right) zone of the diagram.

- A time course assay of the Li coefficients (that are proportional to colocalization rates) throughout the duration time of the experiment, obtained from the average Li values of the 600-900 cells analyzed from each condition.

-  A representative picture of both staining for each condition.

-  A representative plot profile of both antigens' fluorescences showing the colocalization presence or absence using correlation coefficient calculations in relevant fluorochrome 1-positive and fluorochrome 2-positive areas of the representative biological sample.

References

1. Bolte S, Cordelieres FP. A guided tour into subcellular colocalization analysis in light microscopy. Journal of microscopy. 2006;224(Pt 3):213-32. Epub 2007/01/11. doi: 10.1111/j.1365-2818.2006.01706.x. PubMed PMID: 17210054.

2. Li Q, Lau A, Morris TJ, Guo L, Fordyce CB, Stanley EF. A syntaxin 1, Galpha(o), and N-type calcium channel complex at a presynaptic nerve terminal: analysis by quantitative immunocolocalization. The Journal of neuroscience : the official journal of the Society for Neuroscience. 2004;24(16):4070-81. Epub 2004/04/23. doi: 10.1523/JNEUROSCI.0346-04.2004. PubMed PMID: 15102922.
